# Ultrathin‐Gold‐Resonators‐Enabled Bolometers with High Linearity, Responsivity, and Repeatability

**DOI:** 10.1002/advs.202521335

**Published:** 2026-02-24

**Authors:** Jiaqi Wu, Luming Wang, Jing Yu, Chenfei Lv, Ziluo Su, Jiaze Qin, Bo Xu, Kuai Yu, Jiankai Zhu, Zenghui Wang

**Affiliations:** ^1^ Institute of Fundamental and Frontier Sciences University of Electronic Science and Technology of China Chengdu China; ^2^ State Key Laboratory of Radio Frequency Heterogeneous Integration College of Physics and Optoelectronic Engineering Shenzhen University Shenzhen China; ^3^ State Key Laboratory of Precision Measuring Technology and Instruments (Tianjin University) Tianjin China; ^4^ Hubei Key Laboratory of Micro‐Nanoelectronic Materials and Devices Hubei University Wuhan China; ^5^ State Key Laboratory of Electronic Thin Films and Integrated Devices University of Electronic Science and Technology of China Chengdu China

**Keywords:** bolometers, frequency scaling, nanomechanical resonators, ultrathin metal

## Abstract

Ultrathin metal films, with thickness down to just a few nanometers, boast unique optical and electronic properties compared with bulk metals, and show great potential in advanced sensing applications. Incorporating mechanical degrees of freedom would offer a promising route toward further enriching their sensing capabilities, yet this possibility has been rarely explored to date. Here, we demonstrate ultrathin gold nanomechanical resonant sensors with robust vibrations in the high frequency (HF) and very high frequency (VHF) bands. We show that such devices can function as nanoscale bolometers with good linearity (nonlinearity factor of 0.0865), high power‐to‐frequency responsivity (∼−11.47 ppm · µW^−1^), and excellent repeatability across a broad range of laser power (2.3 µW–0.48 mW). We further elucidate the frequency scaling law of these resonant sensors, extracting a Young's modulus of 75.6 GPa for the ultrathin gold crystal and a device pretension of 0.09–0.8 N · m^−1^. Our work paves the way toward future wafer‐scale design and on‐chip integrated sensors based on ultrathin metal nanomechanical devices.

## Graphical abstract

This work demonstrates ultrathin gold nanomechanical resonators operating in HF/VHF bands, unveiling their potential for laser power sensing. By exploiting frequency shifts resulting from laser‐induced thermal tension, these devices can function as sensitive nanoscale bolometers, with outstanding linearity, responsivity, and repeatability. The findings also elucidate the frequency scaling law, offering design guidelines for nanoelectromechanical devices based on ultrathin gold material.

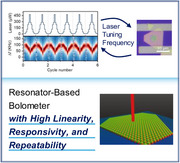

## 1 Introduction

Ultrathin metal films with nanometer‐scale thicknesses have attracted considerable attention, thanks to their distinct optical and electronic properties in contrast to bulk metals. Benefiting from their high surface‐to‐volume ratio [1] and quantum confinement effects [2, 3, 4], ultrathin metals—such as gold films—exhibit tunable electronic band structures [5] and pronounced surface plasmon resonance (SPR) [6, 7, 8, 9, 10, 11]. Moreover, these intriguing ultrathin materials possess exceptional electrical conductivity [12, 13, 14, 15], strong light‐matter interactions [14, 16, 17], high optical transmittance [15], remarkable electro‐optic tunability [18, 19], and superconductivity in the single‐atomic‐layer limit [20, 21].

Building upon these unique capabilities, ultrathin metals have attracted growing interest for sensing applications. For example, gold thin‐film gas sensors exhibit sensitivities more than six times better than those of bulk gold [22], while ultrathin metal strain sensors demonstrate high linearity and outstanding resolution [23], making them suitable for pulse, acoustic, and biomedical signal detection [24, 25, 26]. To further broaden the sensing potential of ultrathin materials, including those based on non‐layered systems [27, 28, 29], one feasible route is to introduce mechanical degrees of freedom [30, 31, 32, 33] such as creating nanoelectromechanical system (NEMS) devices. This approach has been well explored by the applications of low‐dimensional materials such as graphene and carbon nanotubes in NEMS‐based sensors, creating record‐breaking sensing performance in force [34], mass [35, 36], and displacement detection [37]. However, nanoelectromechanical devices based on ultrathin metals remain scarcely studied, leaving their great sensing potential largely unexplored.

In this study, we demonstrate 2D gold‐based nanomechanical resonant sensors, enabled by chemically synthesizing single‐crystal gold flakes with nanoscale thickness. The resulting NEMS resonators exhibit resonance frequencies ranging from 4.6 to 36.5 MHz, spanning the high frequency (HF) and very high frequency (VHF) bands, with displacement noise levels as low as 69 fm · Hz^−1/2^ (See Section S3 for details). We further demonstrate their sensing functionality as bolometers, exhibiting good linearity (nonlinearity factor of 0.0865), high power‐to‐frequency responsivity (∼ −11.47 ppm · µW^−1^), and excellent repeatability across a broad laser power range (2.3 µW – 0.48 mW). In addition, we establish the frequency scaling law for gold‐based NEMS resonators, extracting a Young's modulus of 75.6 GPa and a pretension of 0.09 – 0.8 N · m^−1^. This study not only deepens the fundamental understanding of the mechanical properties of such gold nanostructures, but also paves the way for future gold‐based functional devices and integrated nano‐sensors.

## 2 Results

**FIGURE 1:**
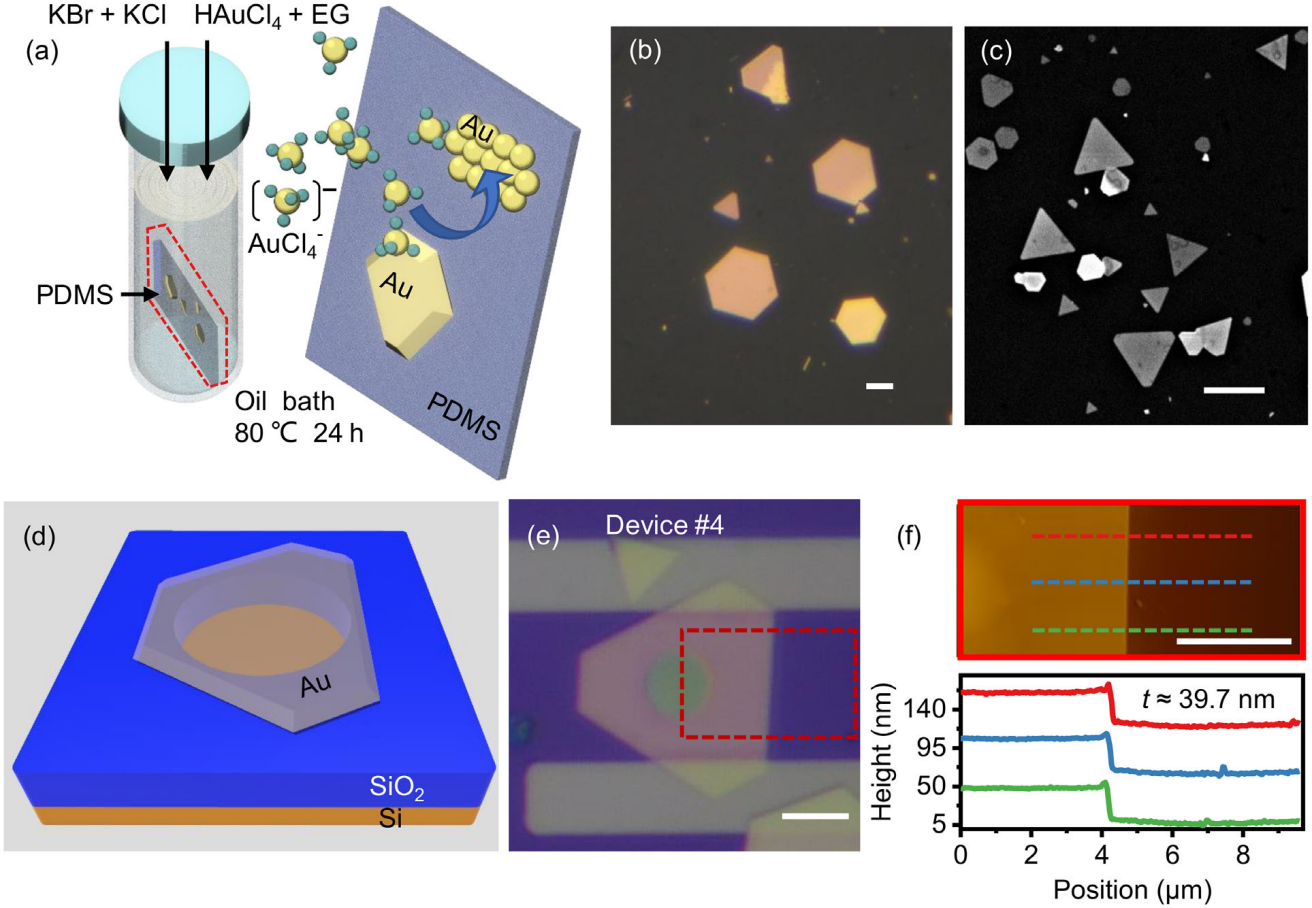
Sample preparation. a) Schematic of the synthesis of ultrathin gold nanoflakes on PDMS in ethylene glycol (EG). b) Optical micrograph and c) SEM image of gold nanoflakes synthesized on PDMS. d) Illustration of a gold nanomechanical resonator, where a piece of gold nanoflake is suspended over a pre‐etched circular hole, forming a suspended circular drumhead. e) Optical image of Device #4. f) AFM image showing the area within the red box in e) and thickness measurements at the positions indicated by the colored dashed lines, with a measured thickness of approximately 39.7 nm. All scale bars: 5 µm.

We fabricate single‐crystal gold nanomechanical resonators using gold nanoflakes synthesized on polydimethylsiloxane (PDMS) substrates (Figure 1a, details in Methods). We select gold flakes suitable for device fabrication on the basis of their lateral dimensions and surface quality, assessed using optical microscopy (Figure 1b) and scanning electron microscopy (SEM, Figure 1c). The selected nanoflakes are then flip‐transferred onto pre‐etched circular microtrenches to form suspended drumhead structures (Figure 1d, details in Methods and Section S1).

We measure the thickness of each resonator using atomic force microscopy (AFM). As a representative device, #4 (optical image in Figure 1e) has a diameter of 5 µm with a smooth (no observable defect) surface and a thickness of 39.7 nm (AFM image in Figure 1f). Interestingly, the suspended gold in Figure 1e appears semitransparent, allowing the underlying circular micro‐trench to be clearly visible. It's important to note that a finite optical transmission through the Au membrane, especially in thinner membranes, is a key enabling factor for subsequent vibration measurements based on optical interferometry. Device dimensions and AFM‐measured thicknesses are summarized in Table S1, with thicknesses ranging from 33.8 nm to 235.3 nm across all the devices.

**FIGURE 2:**
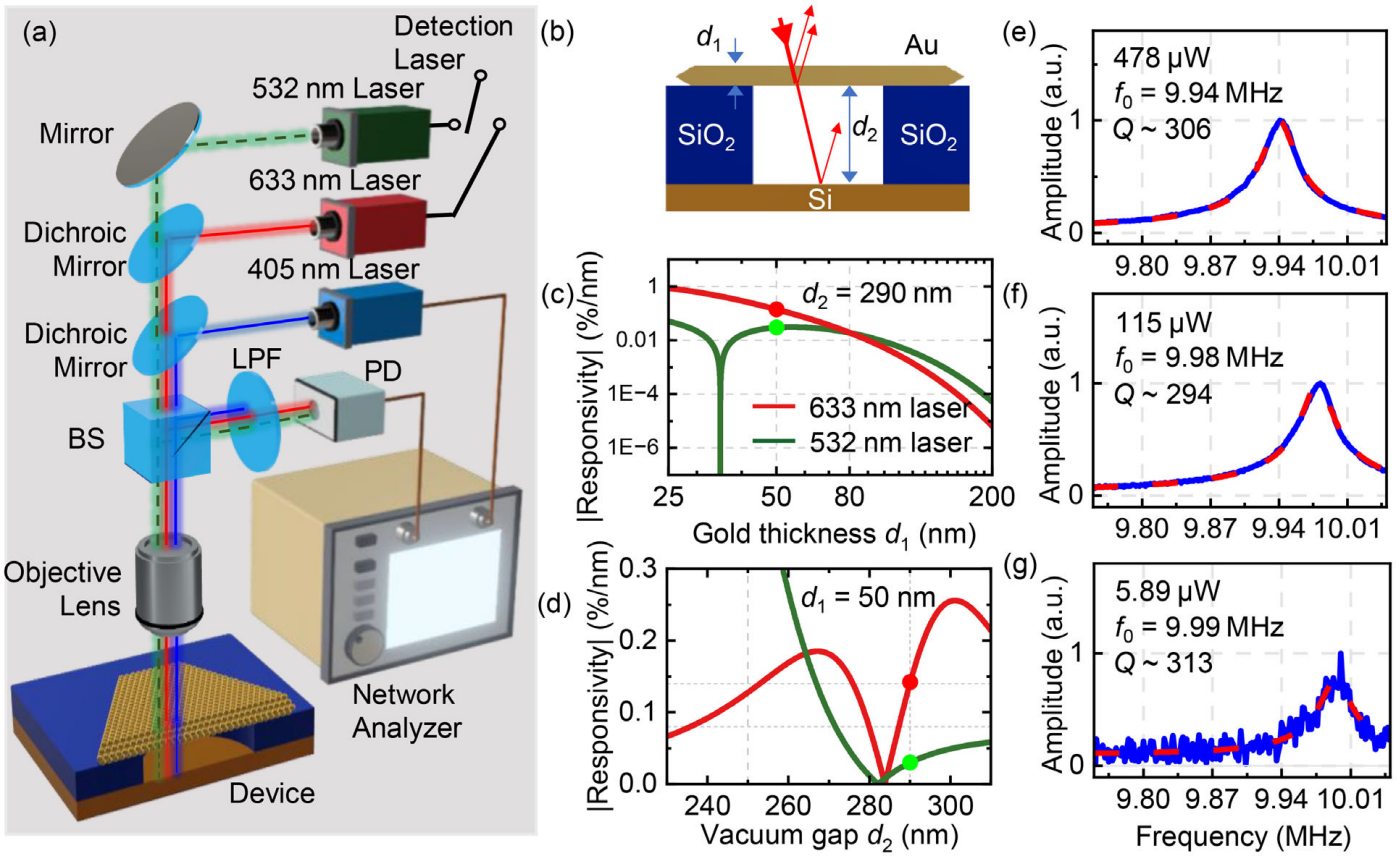
Interferometric measurement of device response. a) Custom‐built interferometry system. PD: photodetector, BS: beam splitter, LPF: long pass filter. b) Schematic diagram of device structure. c) Optical interferometric responsivity versus material thickness *d*_1_, with *d*_2_ = 290 nm. d) Responsivity versus vacuum gap *d*_2_, with *d*_1_ = 50 nm. Red and green lines represent detection lasers at 633 and 532 nm, respectively. The red and green dots in c) and d) represent the device geometry in which the two plots converge. e, f, g) Normalized resonance response (blue) and corresponding fittings (red dashed) under varying detection laser powers.

We employ a custom‐built optical interferometry system (Figure 2a) to measure the vibrational response of ultrathin gold resonators [38], with all measurements performed under high vacuum (∼10^−4^ mbar) at room temperature. To actuate the mechanical vibrations, we modulate the intensity of a 405 nm diode laser with a vector network analyzer (VNA), thereby periodically heating and thus photothermally driving the suspended membrane. To detect the out‐of‐plane motion, we focus a 633 nm (or 532 nm) detection laser onto the device and read out the interferometric signal. Since ultrathin gold is semitransparent, part of the detection laser is reflected at the top surface, while part of it is transmitted through the membrane and then reflected from the other surfaces. The reflected beams from different interfaces (vacuum‐Au, Au‐vacuum, and vacuum‐Si) interfere with one another (Figure S2), allowing the resonant motion to periodically modulate the reflected light intensity. We use a photodetector (PD) to collect the reflected light carrying the vibrational information and convert it into the voltage domain, which is subsequently fed into the vector network analyzer. Based on such a highly sensitive measurement scheme [39, 40, 41], we successfully measure the fundamental‐mode resonance response of 35 gold resonators (full data in Figures S14‐S16).

To optimize signal‐to‐noise ratio (*SNR*) in resonance measurements, we analyze the optical transduction using a Fresnel‐law optical model [42] (details in Methods and Supporting Information). Specifically, for the device structure, we calculate reflectance *R* (details in Section S2) and the ‘displacement‐to‐reflectance’ responsivity ℜ (ℜ = ∂*R*/∂*d*_2_ where *d*_2_ is the vacuum gap) under different device designs and laser wavelengths (633 and 532 nm), with the results shown in Figure 2b–d. Figure 2c shows how ℜ varies with gold flake thickness *d*_1_, with red/green curves for a 633/532 nm laser. Our analysis indicates that optimizing the gold thickness and excitation wavelength can effectively enhance the responsivity, with the 633 nm offering the most favorable performance in the thin‐film regime (*d*_1_ < 80 nm). We also examine how ℜ varies with vacuum gap *d*_2_ (Figure 2d, fixed *d*_1_ = 50 nm), in order to identify the optimal substrate structure for a given gold thickness.

Next, we focus on examining the effect of varying the 633 nm laser power on the resonance response of the gold resonators. Figure 2e–g shows the normalized fundamental‐mode response of the representative Device #4. We fit each spectrum with the simple harmonic oscillator (SHO) model to extract the resonance frequency *f*_0_ and quality factor *Q*. As we vary the 633 nm laser power, *f*_0_ clearly shifts while *Q* remains nearly constant, revealing a pronounced response to laser power. Such responsivity can be attributed to laser‐induced softening [43] (see Section S4 for details).

**FIGURE 3:**
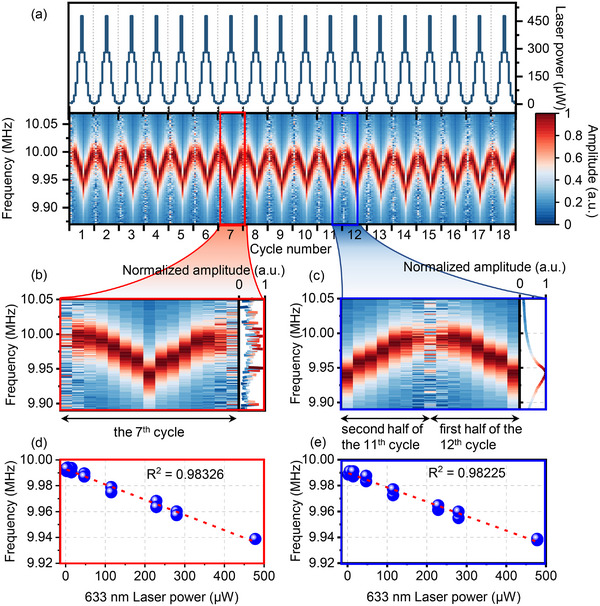
Dynamic response of the bolometer. (a) 2D color plot of the normalized resonance response (bottom) and corresponding detection laser power (top); (b, c) Two selected cycles from (a) with resonance spectra of their respective last measurement on the right. In (b, c), the *x*‐axis corresponds to one complete power cycle; (d, e) Scatter plots of extracted resonance frequency as a function of detection laser power for the cycles in (b, c). Dashed lines represent linear fits with both R^2^ values exceeding 0.98.

To characterize this laser power sensing capability in gold nanomechanical resonators, we vary the 633 nm laser power between 2.3 µW and 0.478 mW, and monitor the resonance response. We repeat this procedure for 18 consecutive power cycles to verify the repeatability of the frequency‐power response, where one cycle corresponds to a complete laser‐power increase‐decrease sequence. Figure 3a presents a 2D color plot of the resonant response (lower panel, red = high amplitude) in relation to laser power cycles (upper panel). To quantify responsivity and linearity, which are two important metrics in sensing applications, we zoom into two cycles (with opposite phases, Figure 3b,c) and examine the corresponding frequency‐power scatter plots (Figure 3d,e, raw data in Figure S8). The data are fitted using a linear model *f*_0_(*P*) = *f*_0_(0) + *kP* (*P*: laser power; *k*: slope), yielding R^2^ values greater than 0.98 for both datasets.

We further calculate the nonlinear factor using Equation (1) [44, 45, 46]:

(1) $$NL_{i}=\frac{\Delta f_{0}\left(P_{i}\right)-\frac{\Delta f_{0,\max}}{\Delta P_{\max}}\left(P_{\max}-P_{i}\right)}{\Delta f_{0,\max}}$$

where *P_i_* is the laser power at the *i*‐th measurement point, ∆P_max_ = P_max_ − P_min_ (P_min_ and P_max_ are the minimum and maximum power (operation range)), ∆*f*_0_(*P_i_*) = *f*_0_(*P_i_*) − *f*_0_(P_max_) and ∆*f*_0,max_ = *f*_0_(P_min_) − *f*_0_(P_max_) (*f*_0_(*P_i_*) is the frequency of corresponding laser power *P_i_*). Using this equation, we obtain an *NL*_max_ of only 0.0865, representing a very small maximum deviation from the linear fit, confirming the highly linear response of the laser power sensor. From the fitted slope *k* of all cycles, we estimate the power‐to‐frequency responsivity to be −114.0 ± 5.5 Hz · µW^−1^, corresponding to a relative frequency shift of approximately −11.47 ± 0.6 ppm · µW^−1^(details in Section S5). This demonstrates that even microwatt‐scale changes in optical power can induce clearly measurable frequency shifts of the device, underscoring the potential of ultrathin gold resonators for high‐performance bolometric sensing.

**FIGURE 4:**
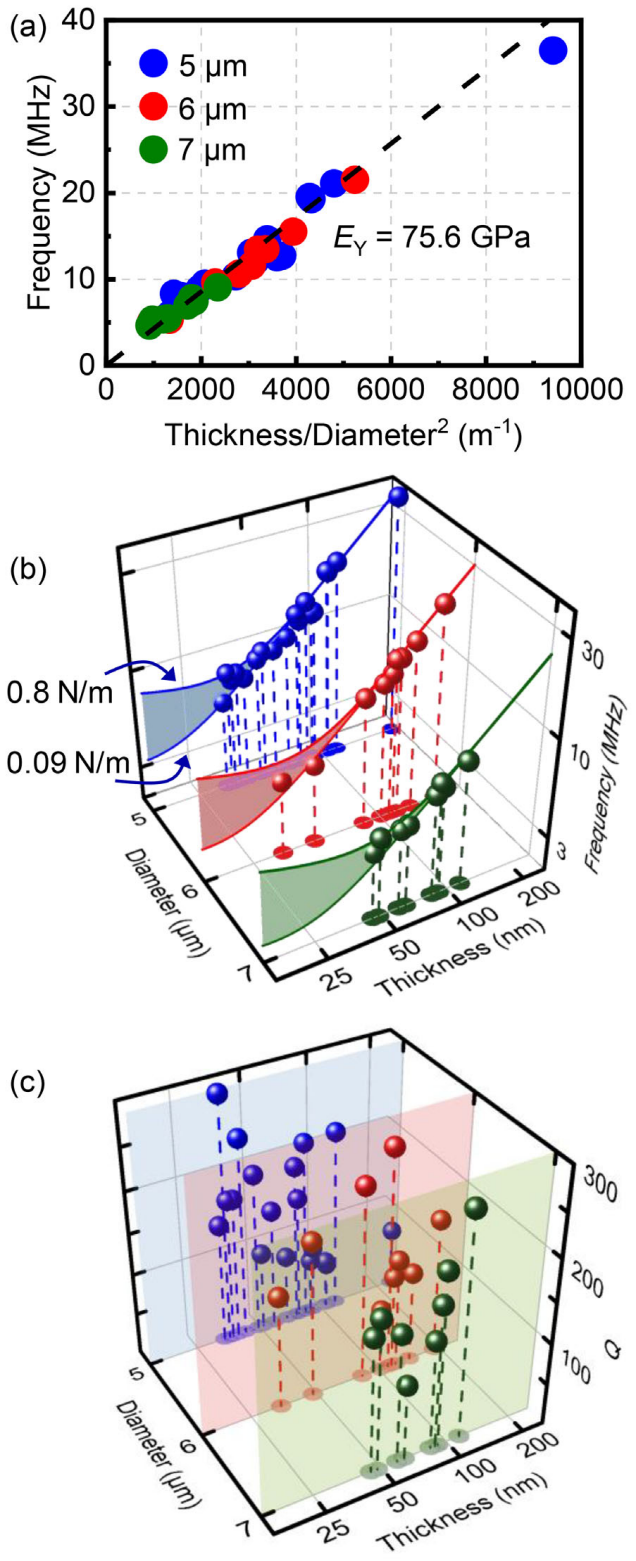
Performance of all the 35 devices. (a) frequency *f*_0_ plotted as a function of thickness‐to‐diameter‐squared ratio (*t*/*a* ^2^). The dashed line represents calculated frequencies from the circular plate model for *E*_Y_ = 75.6 GPa. (b, c) 3D scatter plots of fundamental‐mode resonance frequency *f*_0_ (b) and quality factor *Q* (c) vs. diameter *a* and thickness *t*.

We further investigate the frequency scaling law of ultrathin gold NEMS sensors to establish the device design guidelines and extend their operating frequency ranges. We measure 35 gold nanomechanical resonators with varied diameters and thicknesses, extract *f*_0_ and *Q* for all of them, and plot the results in Figure 4 and summarize in Table S1. Among them, the highest fundamental‐mode frequency‐quality factor product *f*_0_ × *Q* reaches 4.55 × 10^9^ Hz, representing a key performance metric for radio frequency (RF) devices [40]. To elucidate the influence of geometry (diameter *a*, thickness *t*) and material properties (Young's modulus *E*_Y_, Poisson's ratio *ν*, mass density *σ*) on the resonance frequency, we model each resonator as a suspended, fully clamped circular drum. The fundamental‐mode frequency follows [47]:

(2) $$f_{0}=\left(\frac{\mathrm{ka}}{4\pi }\sqrt{\frac{16D}{\sigma_{\mathrm{gold}}a^{4}}\left[{\left(\frac{\mathrm{ka}}{2}\right)}^{2}+\frac{\gamma a^{2}}{4D}\right]}\right)$$

where *σ*_gold_ is the areal mass density of gold, (*ka*) is a mode‐dependent parameter (see Section S7), *γ* is the surface tension of the membrane, and *D* = *E*_Y_ *t* ^3^ / [12(1‐*ν* ^2^)] is the flexural rigidity. In the low‐tension limit *γa* ^2^/(4*D*) → 0, where bending stiffness dominates, the expression simplifies to (details in Section S7):

(3) $$f_{0}\approx \frac{2\beta_{0}}{\pi }\sqrt{\frac{E_{Y}}{12\rho_{\mathrm{gold}}\left(1-\nu^{2}\right)}}\frac{t}{a^{2}}$$

According to Equation (3), the resonance frequency scales linearly with *t*/*a* ^2^, where *ρ*_gold_ is the bulk mass density and *β*_0_ = 10.215 for the fundamental mode [47]. As shown in Figure 4a, the experimental data clearly follow this trend, unambiguously yielding an effective Young's modulus of about 75.6 GPa for ultrathin gold flakes, in good agreement with both the reported value from elastic‐plastic deformation measurements (79 GPa) [48] and theoretical calculations [49, 50, 51].

Figure 4b, c presents the measured fundamental‐mode resonance frequency (*f*_0_) and quality factor (*Q*) of the 35 resonators as functions of device diameter and thickness. The results indicate that ultrathin metal NEMS devices exhibit resonance frequencies exceeding 30 MHz and quality factors above 300 at room temperature. Notably, each solid line in Figure 4b represents the theoretical calculation based on Equation (2), which agrees well with our experimental results. From the data, we further extract the surface tension of ultrathin gold NEMS devices to be in the range of 0.09–0.8 N · m^−1^ (See Section S8 for details), in excellent agreement with previously reported values for 2D NEMS devices [40, 52, 53]. In addition, it is observed that *Q* exhibits an overall trend of increase with thickness, in agreement with theoretical analysis [54].

## 3 Conclusions

In this study, we successfully demonstrate high‐performance NEMS bolometers based on ultrathin gold resonators. Benefiting from a finite optical transmission offered by ultrathin gold nanoflakes, highly sensitive interferometric measurements can be used to detect the infinitesimal nanomechanical motion in these exquisite devices. These resonant nanoscale sensors exhibit good linearity (nonlinearity factor as low as 0.0865), high power‐to‐frequency responsivity (∼ −11.47 ppm · µW^−1^), and excellent repeatability across a broad laser power range (2.3 µW – 0.478 mW). They also exhibit robust performance in the high‐frequency (HF) and very high‐frequency (VHF) bands, spanning from 4.6 to 36.5 MHz. We further elucidate the frequency scaling law in ultrathin gold resonators and extract both the Young's modulus (75.6 GPa) and pretension (0.09–0.8 N · m^−1^) of ultrathin gold crystals. These findings highlight the unique advantages of gold‐based 2D resonant sensors, underscoring their exceptional potential for high‐performance integrated sensing applications.

## 4 Methods

### 4.1 Gold (Au) Synthesis

During the high‐temperature thermal treatment (80°C in oil bath), ethylene glycol (EG, 99.8%) serves as both the solvent for the metal precursor and the reducing agent [55, 56, 57], converting gold ions (100 µL of 0.1 m aqueous solution of HAuCl_4_) into metallic gold. The ‘facet‐selective adsorption hypothesis’ [58] explains the anisotropic growth of gold (Au), where halide ions selectively bind to specific crystal facets, lowering their surface energy (thermodynamic control) or slowing the rate of material addition (kinetic control) compared to other facets. Chloride (Cl^−^) (50 µL of 50 mm aqueous solution of KCl) and bromide (Br^−^) (50 µL of 50 mM aqueous solution of KBr) ions preferentially adsorb onto the top {111} facet of the Au seeds, forming a halide adlayer beneath. This significantly suppresses the growth kinetics of the top Au {111} basal facet while promoting growth along the side facets, which are less covered by the halide ion layer. After a growth period of 24 h, gold nanoflakes of varying thickness with smooth surfaces are obtained.

### 4.2 Device Fabrication

The fabrication process of gold nanomechanical resonators is shown in Figure S1. First, the PDMS pieces with gold nanoflakes grown on them are cut into approximately 5 mm × 5 mm sizes. Next, gold nanomechanical resonators are fabricated by transferring (with alignment) the target gold flakes onto SiO_2_ (290 nm) on Si substrates with prepatterned circular cavities (diameter *a* = 5, 6, 7 µm; cavity depth 290 nm).

### 4.3 Thickness Measurements

AFM measurements are used to determine the flake thickness. The AFM images are taken using a tapping mode AFM with a tip frequency of 290.4556 kHz. The AFM image in Figure 1e (Device #4 in Table S1) is taken with a scan range of 15 µm × 15 µm (the shown area is cropped to 15 µm × 7.4 µm), a resolution of 512 lines and 512 points/line, and a line scan rate of 0.5 Hz. Thicknesses of all other samples in Table S1 are measured with AFM using similar settings.

### 4.4 Responsivity Calculation

The Fresnel‐law optical model is used to calculate reflectance *R* and the displacement‐to‐reflectance responsivity is ℜ = ∂*R*/∂*d*_vac_, where *d*_vac_ is the vacuum gap (see Section S2 for details).

4.4.1

A vertically incident laser beam with intensity *I*_incident_ reflects at each interface within the layered structure. As the 2D resonator is driven into motion, the vacuum gap *d*_vac_ dynamically changes, leading to a periodic modulation of the total reflected light intensity *I*_interferometry_. The total reflectance *R* of the structure (shown in Figure S2b) can be expressed as follows [41, 59]:

(4) $$\begin{array}{c} R=\frac{I_{\mathrm{interferometry}}}{I_{incident}}={\left|\frac{\begin{array}{c} r_{1}+r_{2}e^{-2i\varphi_{1}}+r_{3}e^{-2i(\varphi_{1}+\varphi_{2})}+r_{4}e^{-2i(\varphi_{1}+\varphi_{2}+\varphi_{3})}+r_{1}r_{2}r_{3}e^{-2i\varphi_{2}}\;+r_{1}r_{3}r_{4}e^{-2i\varphi_{3}}+r_{1}r_{2}r_{4}e^{-2i(\varphi_{2}+\varphi_{3})}+r_{2}r_{3}r_{4}e^{-2i(\varphi_{1}+\varphi_{3})} \end{array}}{\begin{array}{c} 1+r_{1}r_{2}e^{-2i\varphi_{1}}+r_{1}r_{3}e^{-2i(\varphi_{1}+\varphi_{2})}+r_{1}r_{4}e^{-2i(\varphi_{1}+\varphi_{2}+\varphi_{3})}+r_{2}r_{3}e^{-2i\varphi_{2}}\;+r_{3}r_{4}e^{-2i\varphi_{3}}+r_{2}r_{4}e^{-2i(\varphi_{2}+\varphi_{3})}+r_{1}r_{2}r_{3}r_{4}e^{-2i(\varphi_{1}+\varphi_{3})} \end{array}}\right|}^{2} \end{array}$$

where ***r***_1_, ***r***_2_, ***r***_3_ and ***r***_4_ are reflection coefficients at the vacuum‐gold, gold‐vacuum, vacuum‐SiO_2_ and SiO_2_‐Si interface, respectively. And *φ*_1_, *φ*_2,_ and *φ*_3_ are phase shifts that arise from variations in the optical path length:

(5) $$\begin{array}{ccc} & & r_{1}=\frac{n_{\mathrm{vac}}-n_{\mathrm{Au}}}{n_{\mathrm{vac}}+n_{\mathrm{Au}}},r_{2}=\frac{n_{\mathrm{Au}}-n_{\mathrm{vac}}}{n_{\mathrm{Au}}+n_{\mathrm{vac}}},r_{3}\\ & & =\frac{n_{\mathrm{vac}}-n_{\mathrm{Si}O_{2}}}{n_{\mathrm{vac}}+n_{\mathrm{Si}O_{2}}},r_{4}=\frac{n_{\mathrm{Si}O_{2}}-n_{\mathrm{Si}}}{n_{\mathrm{Si}O_{2}}+n_{\mathrm{Si}}} \end{array}$$

(6) $$\varphi_{1}=\frac{2\pi n_{\mathrm{Au}}d_{\mathrm{Au}}}{\lambda },\varphi_{2}=\frac{2\pi n_{\mathrm{vac}}d_{\mathrm{vac}}}{\lambda },\varphi_{3}=\frac{2\pi n_{\mathrm{Si}O_{2}}d_{\mathrm{Si}O_{2}}}{\lambda }$$

According to this calculation and Figure 2c, a 633 nm laser is employed to characterize thinner devices, whereas a 532 nm laser is used for thicker devices.

## Conflicts of Interest

The authors declare no conflict of interest.

## Supporting information




**Supporting File**: advs73788‐sup‐0001‐SuppMat.pdf.

## Data Availability

The data that support the findings of this study are available from the corresponding author upon reasonable request.
