# Supplementary material for: Ultrathin‐Gold‐Resonators‐Enabled Bolometers with High Linearity, Responsivity, and Repeatability
Source: Adv Sci (Weinh). 2026 Feb 24;13(18):e21335. doi: 10.1002/advs.202521335 (PMC13042848; doi:10.1002/advs.202521335)
Supplement: Supplementary file 1 — Supporting File: advs73788‐sup‐0001‐SuppMat.pdf. [file ADVS-13-e21335-s001.pdf]

# Supporting Information

# Ultrathin-Gold-Resonators-Enabled Bolometers with High Linearity, Responsivity, and Repeatability

Jiaqi Wu[1,#], Luming Wang[1,#], Jing Yu[2], Chenfei Lv[1], Ziluo Su[1], Jiaze Qin[1], Bo Xu[1,4,5]*, Kuai Yu[2,]*, Jiankai Zhu[1,]*, Zenghui Wang[1,3]*

**Affiliation:**

[1]Institute of Fundamental and Frontier Sciences, University of Electronic Science and Technology of China, Chengdu 610054, China

[2]State Key Laboratory of Radio Frequency Heterogeneous Integration, College of Physics and Optoelectronic Engineering, Shenzhen University, Shenzhen 518060, China

[3]State Key Laboratory of Electronic Thin Films and Integrated Devices, University of Electronic Science and Technology of China, Chengdu 611731, China

[4]State Key Laboratory of Precision Measuring Technology and Instruments (Tianjin University), Tianjin 300350, China

[5]Hubei Key Laboratory of Micro-Nanoelectronic Materials and Devices, Hubei University, Wuhan 430062, China

**Email:**

Bo Xu: bo_xu@uestc.edu.cn,

Kuai Yu: kyu@szu.edu.cn,

Jiankai Zhu: zhujiankai@uestc.edu.cn,

Zenghui Wang: zenghui.wang@uestc.edu.cn.

[#]These authors contributed equally

[*]Corresponding authors

**S1. Fabrication of Ultrathin Gold Nanoelectromechanical Devices** **1**

**S2. Interferometric Readout of Nanomechanical Vibration** **2**

**S3. Measurement and Analysis of Thermomechanical Motion and Displacement Sensitivity** **6**

**S4. Analysis of Optothermal Tuning** **8**

**S5. Linear Fitting with Different Sampling Strategies and Estimation of Average Responsivity** **12**

**S6. Analysis of Thermal Equilibrium Establishment Process under Laser Power Modulation** **13**

**S7. Theoretical Analysis of Device Elastic Behavior** **17**

**S8. Extraction of Tension $\gamma$ from Resonance Measurement** **19**

**S9. Frequency Response of Different Devices** **21**

**References** **27**

## S1. Fabrication of Ultrathin Gold Nanoelectromechanical Devices

We fabricate ultrathin gold nanomechanical resonators using a dry-transfer technique, a well-established method for transferring two-dimensional (2D) materials. We first grow single-crystalline gold nanoflakes directly on a polydimethylsiloxane (PDMS) substrate by wet-chemical synthesis. After synthesis, we cut the PDMS into 5 mm × 5 mm squares and use optical microscopy to roughly estimate the lateral size and thickness of the nanoflakes. We then place each PDMS piece, with the gold side facing up, onto a glass slide, and then invert the slide to align the selected nanoflake over a pre-patterned circular microtrench (depth: 290 nm). Once aligned, we gently bring the nanoflake into contact with the patterned substrate, where it adheres through van der Waals interactions.

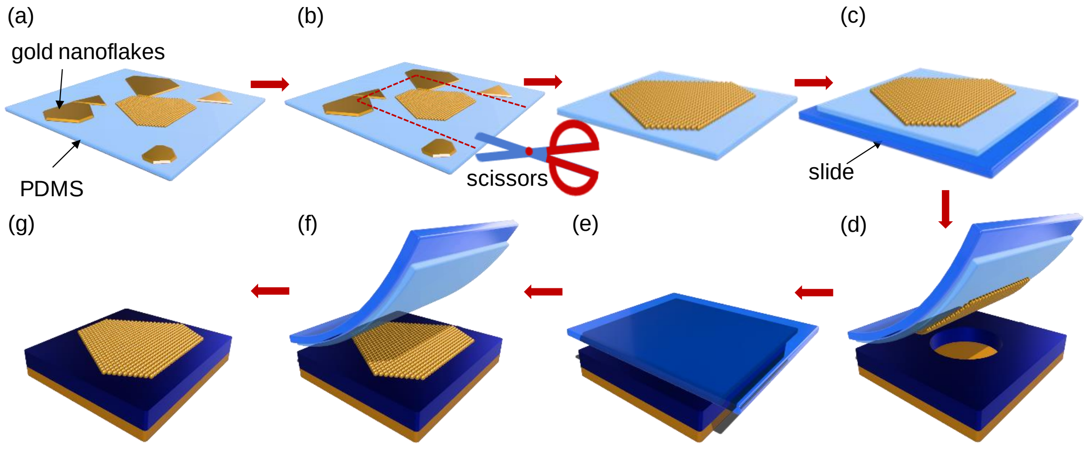


**Figure S1 Fabrication of gold nanomechanical resonators.** (a) Gold nanoflakes grown on a PDMS substrate. (b) The PDMS substrate is cut into approximately 5 × 5 mm squares to match the target substrate size. (c) A PDMS film containing a selected nanoflake is mounted on a glass slide. (d-g) The gold crystals are dry-transferred onto a pre-patterned microtrench substrate. The curvature in the glass slide is only for illustrative purposes (to show what's underneath); in actual experiments, the glass slide remains flat.

## S2. Interferometric Readout of Nanomechanical Vibration

We detect the device motion using laser interferometry (Figure S2). As the ultrathin gold drumhead vibrates, the vacuum gap $d_{\mathrm{vac}}$ ($d_2$ in main text) periodically changes, modulating the total reflected light intensity. This infinitesimal motion is detected via ultrasensitive optical interferometry, achieving a displacement sensitivity on the order of $\mathrm{fm/Hz}^{1/2}$.

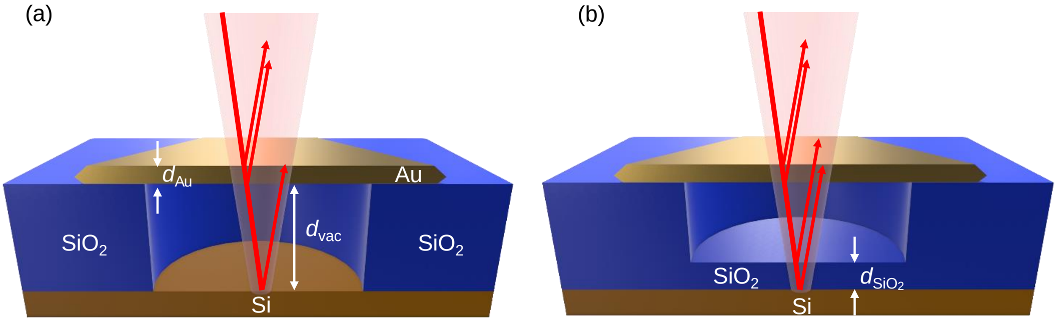


**Figure S2 Schematics of laser interference for two different structures.** The incident laser reflects at all interfaces: (a) vacuum/Au, Au/vacuum, vacuum/Si; (b) vacuum/Au, Au/vacuum, vacuum/$SiO_2$, $SiO_2$/Si.

To illustrate the calculation of the total reflectance $R$, we consider the multilayer structure shown in Figure S2(b). $R$ is defined as the ratio of the total (from this multilayer structure) reflected light intensity $I_{\mathrm{interferometry}}$ to the incident intensity $I_{\mathrm{incident}}$[2,3]:

$$R = \frac{I_{\mathrm{interferometry}}}{I_{incident}} = \left| \frac{\begin{array}{l} \boldsymbol{r}_1 + \boldsymbol{r}_2 e^{-2i\varphi_1} + \boldsymbol{r}_3 e^{-2i(\varphi_1+\varphi_2)} + \boldsymbol{r}_4 e^{-2i(\varphi_1+\varphi_2+\varphi_3)} + \boldsymbol{r}_1\boldsymbol{r}_2\boldsymbol{r}_3 e^{-2i\varphi_2} \\ + \boldsymbol{r}_1\boldsymbol{r}_3\boldsymbol{r}_4 e^{-2i\varphi_3} + \boldsymbol{r}_1\boldsymbol{r}_2\boldsymbol{r}_4 e^{-2i(\varphi_2+\varphi_3)} + \boldsymbol{r}_2\boldsymbol{r}_3\boldsymbol{r}_4 e^{-2i(\varphi_1+\varphi_3)} \end{array}}{\begin{array}{l} 1 + \boldsymbol{r}_1\boldsymbol{r}_2 e^{-2i\varphi_1} + \boldsymbol{r}_1\boldsymbol{r}_3 e^{-2i(\varphi_1+\varphi_2)} + \boldsymbol{r}_1\boldsymbol{r}_4 e^{-2i(\varphi_1+\varphi_2+\varphi_3)} + \boldsymbol{r}_2\boldsymbol{r}_3 e^{-2i\varphi_2} \\ + \boldsymbol{r}_3\boldsymbol{r}_4 e^{-2i\varphi_3} + \boldsymbol{r}_2\boldsymbol{r}_4 e^{-2i(\varphi_2+\varphi_3)} + \boldsymbol{r}_1\boldsymbol{r}_2\boldsymbol{r}_3\boldsymbol{r}_4 e^{-2i(\varphi_1+\varphi_3)} \end{array}} \right|^2, \quad \text{(S1)}$$

where $\boldsymbol{r}_1$, $\boldsymbol{r}_2$, $\boldsymbol{r}_3$ and $\boldsymbol{r}_4$ are reflection coefficients at the vacuum-gold, gold-vacuum, vacuum-$SiO_2$ and $SiO_2$-Si interfaces, respectively. Additionally, $\varphi_1$, $\varphi_2$ and $\varphi_3$ are phase shifts that arise from variations in the optical path length:

$$\boldsymbol{r}_1 = \frac{n_{\mathrm{vac}} - \boldsymbol{n}_{\mathrm{Au}}}{n_{\mathrm{vac}} + \boldsymbol{n}_{\mathrm{Au}}}, \quad \boldsymbol{r}_2 = \frac{\boldsymbol{n}_{\mathrm{Au}} - n_{\mathrm{vac}}}{\boldsymbol{n}_{\mathrm{Au}} + n_{\mathrm{vac}}}, \quad \boldsymbol{r}_3 = \frac{n_{\mathrm{vac}} - \boldsymbol{n}_{\mathrm{SiO_2}}}{n_{\mathrm{vac}} + \boldsymbol{n}_{\mathrm{SiO_2}}}, \quad \boldsymbol{r}_4 = \frac{n_{\mathrm{SiO_2}} - \boldsymbol{n}_{\mathrm{Si}}}{n_{\mathrm{SiO_2}} + \boldsymbol{n}_{\mathrm{Si}}}, \quad \text{(S2)}$$

$$\varphi_1 = \left(2\pi \boldsymbol{n}_{\mathrm{Au}} d_{\mathrm{Au}}\right)/\lambda,\ \varphi_2 = \left(2\pi n_{\mathrm{vac}} d_{\mathrm{vac}}\right)/\lambda,\ \varphi_3 = \left(2\pi \boldsymbol{n}_{\mathrm{SiO_2}} d_{\mathrm{SiO_2}}\right)/\lambda. \quad \text{(S3)}$$

Here, $\boldsymbol{n}$ is the complex refractive index ($n_{vac}$ = 1, $\boldsymbol{n}_{Au}$, $\boldsymbol{n}_{SiO2}$ and $\boldsymbol{n}_{Si}$ stand for the complex refractive indexes of gold, silicon oxide and silicon, respectively). $\lambda$ is the laser wavelength, $d_{Au}$ is the gold membrane thickness, $d_{vac}$ represents the vacuum gap depth, and $d_{SiO2}$ is the silicon oxide thickness. When assuming $d_{SiO2}$ = 0 nm, the formula transforms into a Figure S2(a) structure.

A photodetector continuously monitors changes in reflectance $R$ induced by the resonator's vibration. The optical readout is characterized by the displacement-to-reflectance responsivity $\Re = \partial R/\partial d_{vac}$, which quantifies how $R$ responds to variations in the vacuum gap. A higher $\Re$ yields a larger reflectance change for the same displacement and therefore a stronger signal.

**A. $d_{SiO2}$ = 0 nm**

We consider a Figure S2(a) structure with a gold film thickness of $d_{Au}$ = 50 nm and no silicon oxide layer ($d_{SiO2}$ = 0 nm). Based on Equation S1, we plot the reflectance as a function of the vacuum gap thickness $d_{vac}$ (Figure S3(a)), and plot the optical responsivity $\Re$ as a function of the gold thickness (Figure S3(b)). Additionally, we investigate the variation of $\Re$ with respect to both $d_{Au}$ and $d_{vac}$ under different detection laser wavelengths, as illustrated in Figure S3(c) and Figure S3(d).

**B. $d_{SiO2} \neq 0$ nm**

For the Figure S2(b) structure, we assume $d_{Au}$ = 50 nm and $d_{SiO2}$ = 40 nm. Using Equation S1, we also plot the reflectance as a function of $d_{vac}$ (Figure S4(a)), and plot the optical responsivity $\Re$ as a function of the gold thickness (Figure S4(b)). We further analyze the dependence of $\Re$ on $d_{Au}$ and $d_{vac}$ under various detection laser wavelengths, as shown in Figure S4(c) and Figure S4(d).

| $\lambda$ (nm) | $\boldsymbol{n}_{Au}$ [4] | $\boldsymbol{n}_{SiO2}$ [5] | $\boldsymbol{n}_{Si}$ [6] |
|---|---|---|---|
| 532 | 0.47637 – 2.3579i | 1.46 – 0.0019i | 4.15 – 0.044i |
| 633 | 0.19404 – 3.5934i | 1.46 – 0.016i | 3.881 – 0.019i |

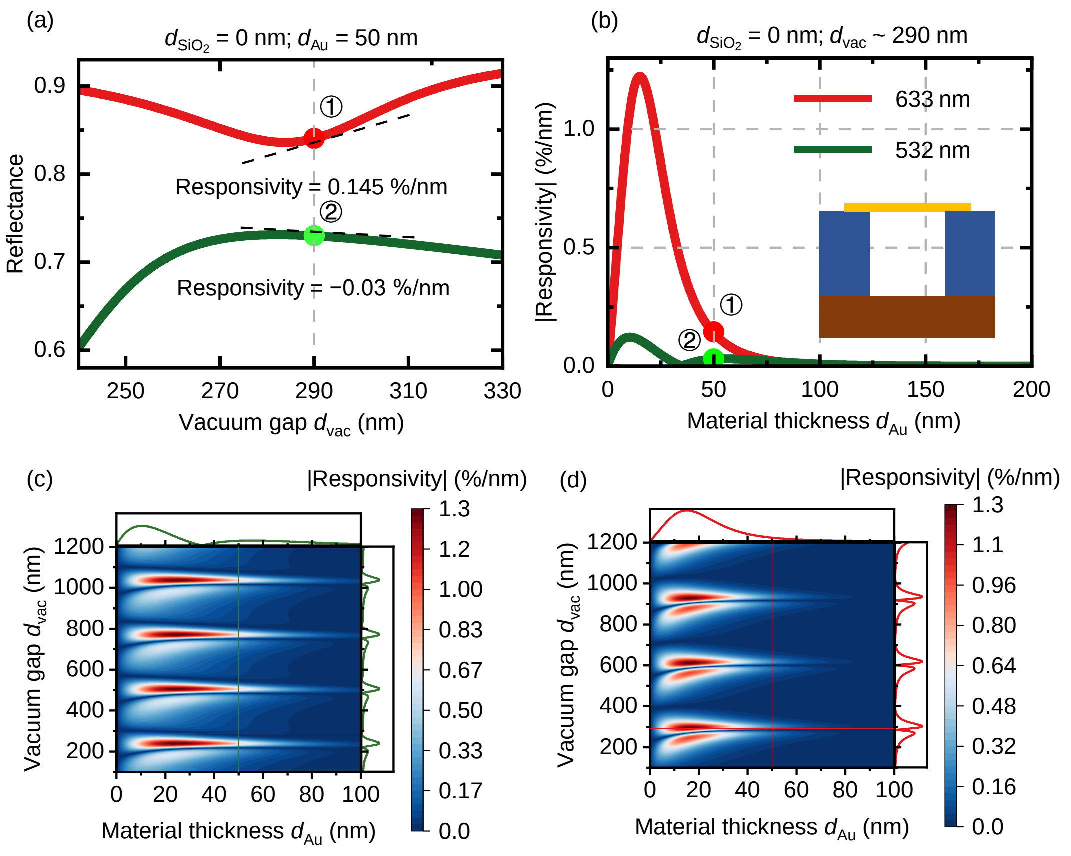


**Figure S3 Optical reflectance and responsivity for the device structure in Figure S2(a).** (a) Optical reflectance of the device as a function of vacuum gap depth for $\lambda$ = 633 nm (red), $\lambda$ = 532 nm (green) and $d_{Au}$ = 50 nm. The slope of the curve at 290 nm (the designed vacuum gap size, indicated by the vertical dashed line) corresponds to the responsivity of the structure in Figure S2(a). (b) Calculated optical interferometric transduction responsivity as a function of gold thickness. (c-d) 2D color plots of responsivity as functions of vacuum gap and gold thickness for (c) $\lambda$ = 532 nm and (d) $\lambda$ = 633 nm.

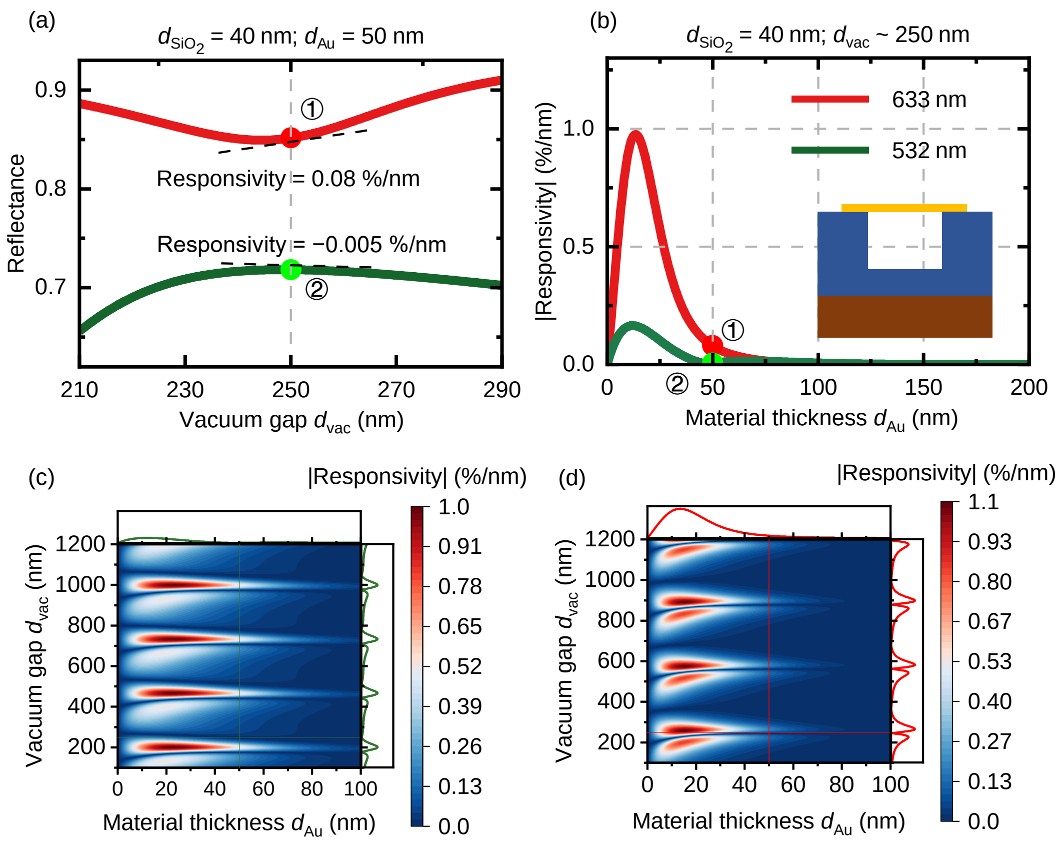


**Figure S4 Optical reflectance and responsivity for the device structure in Figure S2(b).** (a) Optical reflectance as a function of vacuum gap for $\lambda$ = 633 nm (red), $\lambda$ = 532 nm (green), $d_{Au}$ = 50 nm and $d_{SiO2}$ = 40 nm. The slope of the curve at 250 nm (the designed vacuum gap, indicated by the vertical dashed line) corresponds to the responsivity of the structure in Figure S2(b). (b) Calculated optical interferometric transduction responsivity as a function of gold thickness. (c-d) 2D color plots of responsivity as functions of vacuum gap and gold thickness for (c) λ = 532 nm and (d) λ = 633 nm.

## S3. Measurement and Analysis of Thermomechanical Motion and Displacement Sensitivity

We use a spectrum analyzer to measure the undriven resonance response of the device (Figure S5(a)). One such example is shown Figure S5(b), which plots the power spectral density (PSD) in the voltage domain on the left *y*-axis. To relate this electronic signal to the mechanical motion of the resonator, we analyze its thermomechanical noise.

In the frequency domain, the thermomechanical motion of a resonator follows [1,7]:

$$S_{x,th}^{1/2}(\omega) = \left[ \frac{4\omega_0 k_B T}{QM_{eff}} \cdot \frac{1}{(\omega_0^2 - \omega^2)^2 + (\omega_0 \omega / Q)^2} \right]^{1/2}. \quad \text{(S4)}$$

At the resonance frequency, this expression simplifies to:

$$S_{x,th}^{1/2}(\omega_0) = \sqrt{\frac{4k_B TQ}{\omega_0^3 M_{eff}}}. \quad \text{(S5)}$$

Here, $\omega_0$, $K_B$, $T$, $Q$, and $M_{eff}$ denote the angular resonance frequency, Boltzmann's constant, temperature, quality factor, and effective mass, respectively. For the fundamental out-of-plane mode, the effective mass is $M_{eff}$ = 0.1828$M$, where $M$ is the total mass of the resonator [1,8].

For the spectrum shown in Figure S5(b), measured from the 39.7 nm-thick gold resonator, we determine its on-resonance thermomechanical displacement noise spectral density by substituting the measured resonance frequency, quality factor, device dimensions, temperature (300K), and material density into Equation S5. This calculation yields $S_{x,th}^{1/2}$ = 44.17 fm/Hz$^{1/2}$.

Assuming all noise processes are uncorrelated, the total noise PSD equals the sum of the PSDs from individual noise sources. Thus, $S_{v,total}^{1/2} = (S_{v,th} + S_{v,sys})^{1/2}$, where $S_{v,th}^{1/2}$ is the thermomechanical motion noise translated into the voltage domain through the

'displacement-to-voltage' responsivity $\Re \equiv S_{v,th}^{1/2}/S_{x,th}^{1/2}$, and $S_{v,sys}^{1/2}$ is the voltage noise floor of the measurement system that sets the off-resonance background. We then fit the measured noise spectrum using

$$S_{v,total}^{1/2} = \left(\Re^2 \times S_{x,th} + S_{v,sys}\right)^{1/2} = \sqrt{\Re^2\left(\frac{4\omega_0 k_B T}{QM_{eff}} \cdot \frac{1}{(\omega_0^2-\omega^2)^2+(\omega_0\omega/Q)^2}\right)+S_{v,sys}} \ .\text{(S6)}$$

From the fit we extract $Q$ = 89, resonance frequency $f_0$ = 10.25 MHz, $S_{v,sys}^{1/2}$ = 0.233 μV/Hz$^{1/2}$, and $\Re$ = 3.37 μV/pm. We convert the voltage spectral density to displacement spectral density using $S_x^{1/2} = S_v^{1/2}/\Re$ and display this displacement scale on the right y-axis of Figure S5(b). The displacement sensitivity of the measurement system is defined as $S_{x,sys}^{1/2} = S_{v,sys}^{1/2}/\Re$. For the measurement in Figure S5(b), the displacement sensitivity at the resonance frequency is $S_{x,sys}^{1/2}$ = 69 fm/Hz$^{1/2}$.

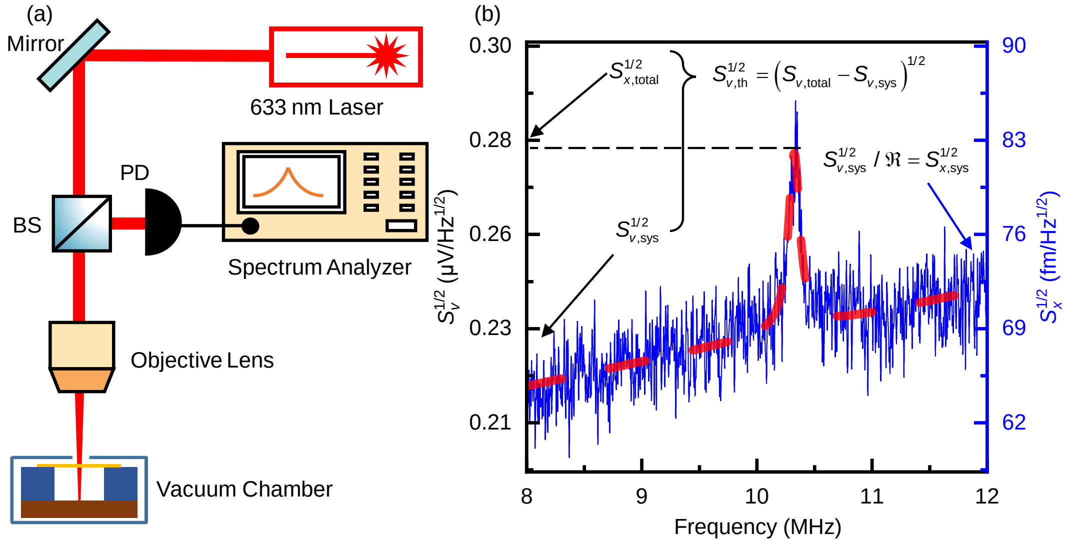


**Figure S5 Thermomechanical motion measurement.** (a) Schematic of the custom-built interferometry setup used together with a spectrum analyzer to measure the undriven thermomechanical motion of the resonator. (b) Representative noise spectrum measured from the device. The annotations show how key quantities can be read off from the spectrum and how they are related to each other.

## S4. Analysis of Optothermal Tuning

The observed power-induced frequency tuning is attributed to laser-induced thermal effects. As the laser power increases, the gold membrane absorbs the incident light, leading to localized heating and subsequent thermal expansion. The resulting fundamental frequency $f_0$ can be expressed as[8,9,10,11]:

$$f_0 = \left(\frac{ka}{4\pi}\right)\sqrt{\frac{16D}{\sigma_{\text{gold}}a^4}\left[\left(\frac{ka}{2}\right)^2 + \frac{(\gamma_0 + \gamma_{\text{th}})\,a^2}{4D}\right]}, \qquad \text{(S7)}$$

where $\gamma_0$ (in N·m$^{-1}$) is the initial surface tension of the membrane, $\gamma_{\text{th}}$ is the laser-induced thermal stress ($\gamma_{\text{th}} < 0$), $\sigma_{\text{gold}}$ (in kg·m$^{-2}$) is the areal mass density, defined as $\sigma_{\text{gold}} = \rho_{\text{gold}} \cdot t$ ($\rho_{\text{gold}}$ = 19.3 g/cm$^3$ is the bulk mass density[12]), $a$ is the diameter of circular drumhead, and ($ka$) is a mode-dependent parameter that can be approximated analytically (see Section S7 for details) [13]. $D = E_Y t^3/[12(1-v^2)]$ is the flexural rigidity ($E_Y$, Young's modulus; $t$: Thickness of gold flakes; $v$: Poisson's ratio, for gold $v = 0.5$)[14].

According to the above model, an increase in laser power causes the gold drumhead to expand, and thus the thermally induced stress change $\gamma_{\text{th}}$ is negative, thereby reducing the total membrane tension ($\gamma_0 + \gamma_{\text{th}}$). As a result, the resonance frequency $f_0$ decreases, consistent with our experimental observations.

As shown in Equation S7, the resonance frequency is collectively determined by the strain and the bending rigidity. Since the bending rigidity scales with the cube of the thickness, how much tension can affect the total frequency also depends on thickness. Qualitatively, frequency of thinner membranes is more responsive to tension, whereas thicker membranes are increasingly dominated by bending rigidity and thus are less affected by tension.

To experimentally show this, we measure the laser-power-induced frequency response of the thickest gold nanomechanical resonator in this work (Device #18), as

shown in Figure S6. In this thickness range, gold exhibits low optical responsivity at 633 nm (as predicted in Fig. 2(c)), so higher laser powers are required to generate measurable resonance signals. We measure the resonance response of this device over a laser power range of 115–478 μW and find that its resonance frequency remains nearly unchanged. This observation supports our hypothesis that thicker membranes are much less sensitive to laser-induced thermal tuning because of their frequency being dominated by bending rigidity.

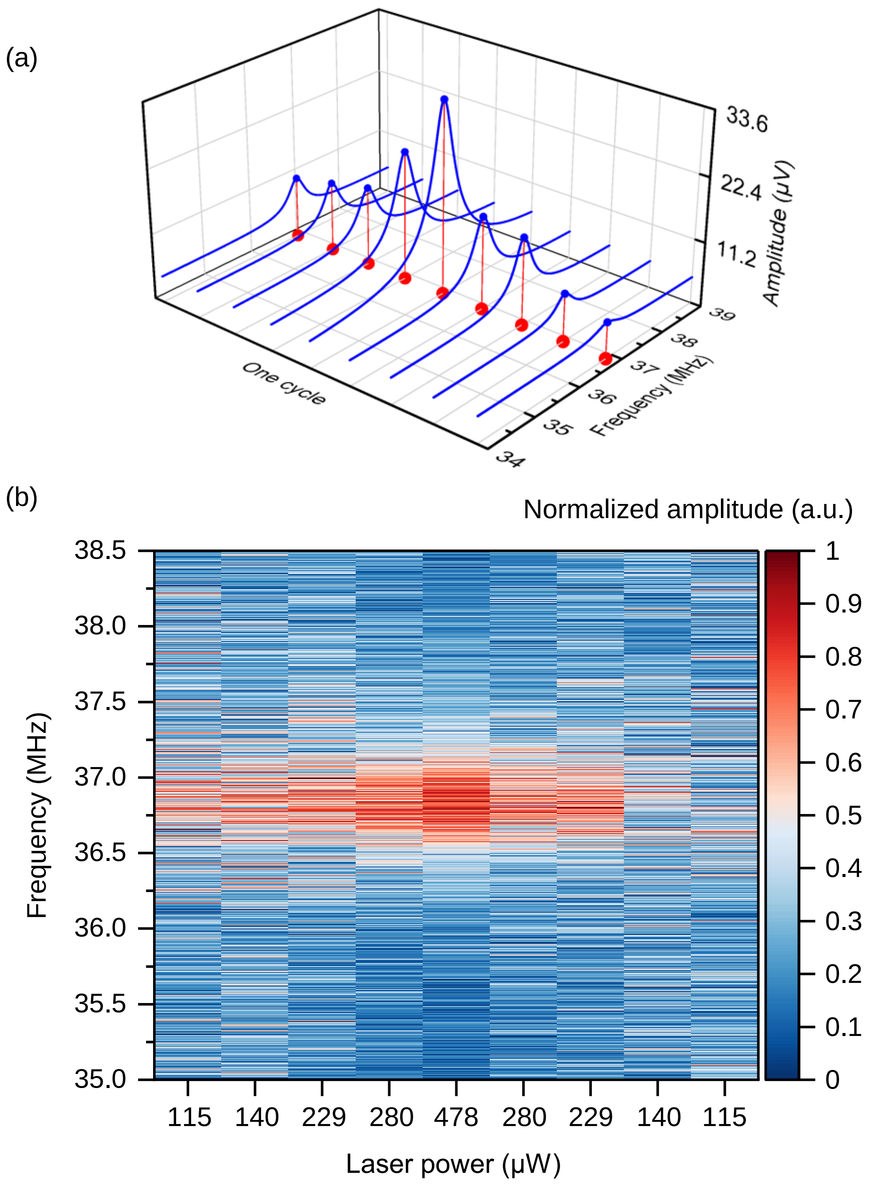


**Figure S6 Resonance frequency variation with laser power for Device #18.** (a) 3D plot of the resonance response, with curves representing the fitted data. The laser power level for each

resonance curve can be found in (b). (b) 2D color map of the normalized resonance response.

In contrast, Figures S7 and S8 show the optothermal tuning behavior of a thinner gold resonator (Device #4). Figure S7 illustrates the time evolution of the normalized resonance response for the first measurement cycle in Figure 3(a), visualized as a 2D color map plotted against the actual measurement time. Figure S8 presents the power-dependent resonance response of the same device, which exhibits a clear frequency shift, demonstrating a pronounced optothermal tuning effect.

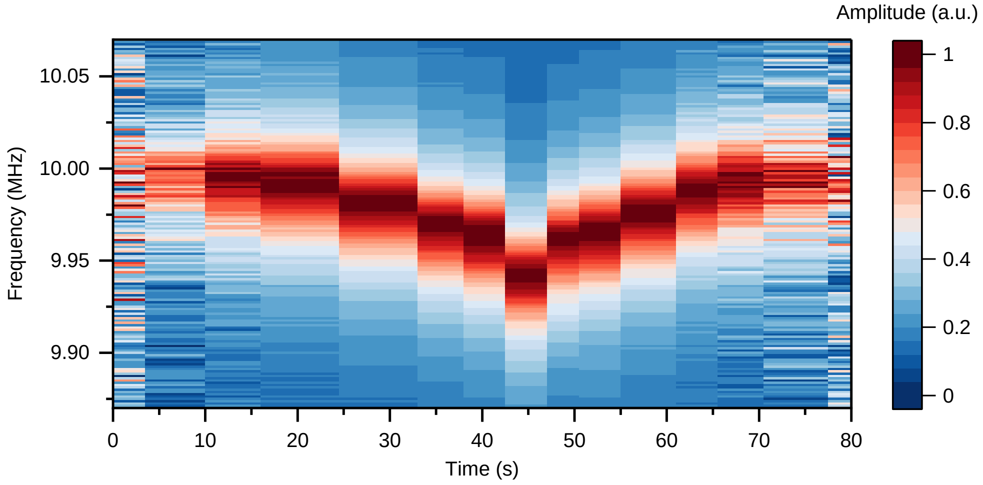


**Figure S7 2D color plot of the normalized resonance response over the actual measurement time, The data range corresponds to the first cycle in Figure 3(a).**

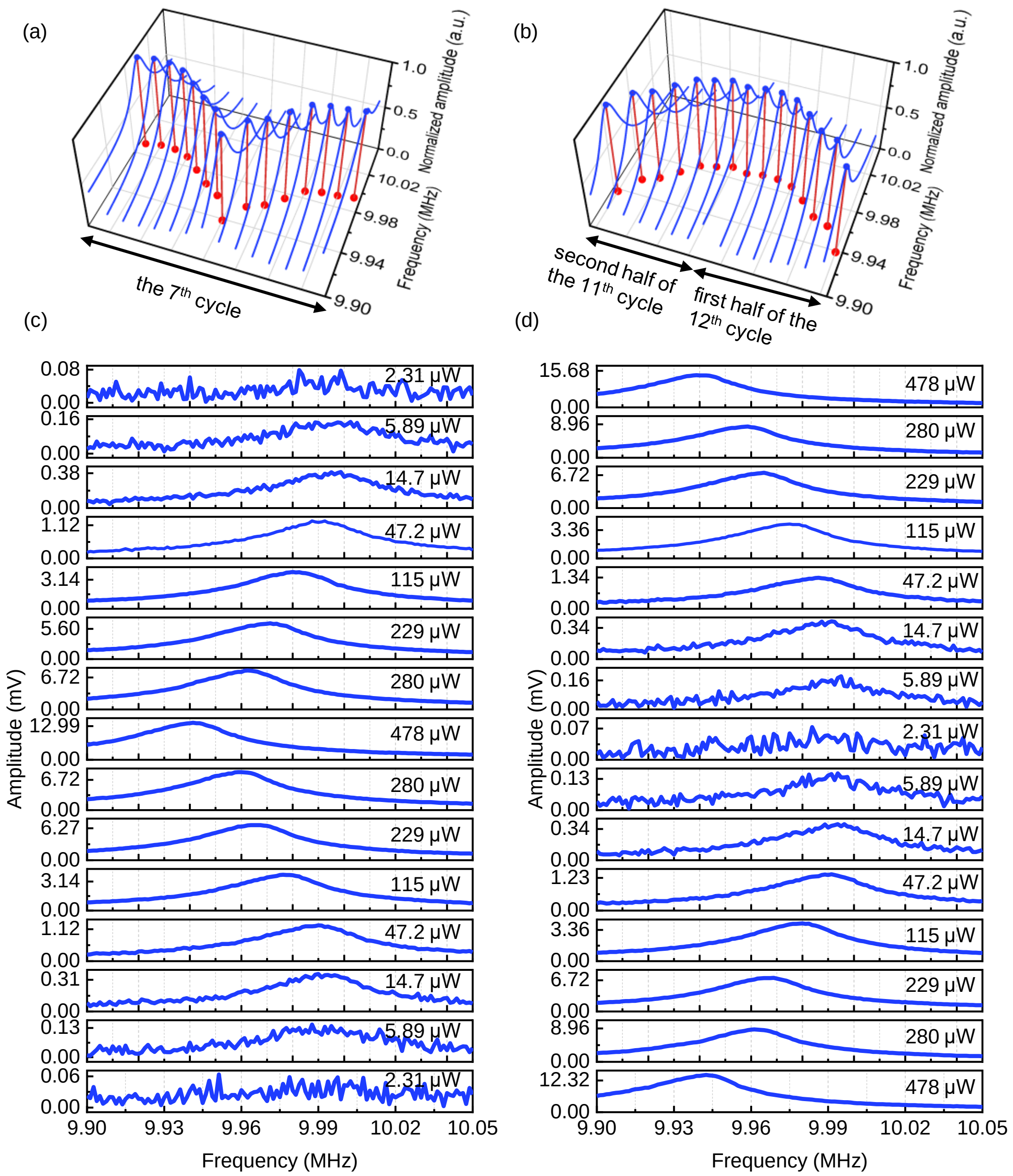


**Figure S8 Evolution of resonance response of the gold resonator during two representative cycles.** (a-b) 3D plots of Figure 3(b) and Figure 3(c), respectively, with the curves representing the fitted data. (c-d) Original spectral data.

## S5. Linear Fitting with Different Sampling Strategies and Estimation of Average Responsivity

To assess how data-point density influences linear fitting and to verify the robustness of the extracted slope *k*, we perform linear fits on the data in Figure 3(d) using different sampling densities. As illustrated in Figure S9, we analyze four different choice of power values that include all points, four points, three points, and two points (Fig. S9). While all the choices of points result in very similar values, to further minimized the potential bias due to the denser sampling in the low-power region, we pick four roughly evenly-spaced power values in each measurement cycle, as in Figure S9(b), to calculate the average of the resulting slopes. This analysis yields an average power-to-frequency responsivity of −114.0±5.5 Hz·μW$^{-1}$, corresponding to a relative frequency shift of −11.47±0.6 ppm·μW$^{-1}$. Note that all the *k* values obtained from different choices of points in Figure S9 fall within this range, confirming the consistency and reliability of the fitting results.

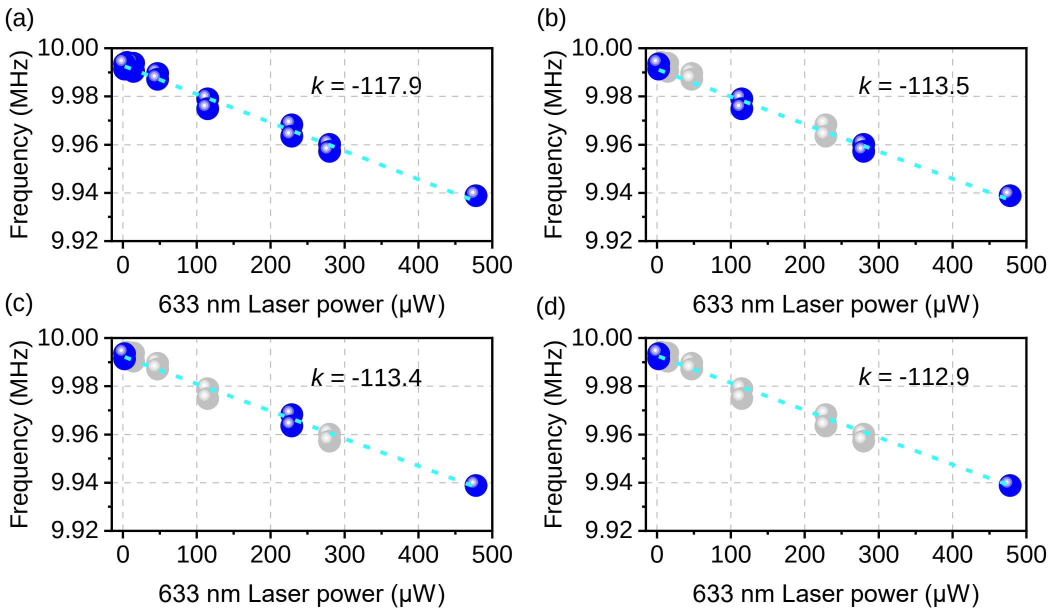


**Figure S9 Linear fitting results with different sets of equally spaced power levels, corresponding to Figure 3(d).** Light grey spheres indicate excluded points, and the fit (blue line) is performed using the remaining data points (blue spheres).

## S6. Analysis of Thermal Equilibrium Establishment Process under Laser Power Modulation

In this section, we present a quantitative analysis of the time scale required to establish thermal equilibrium following a change in incident laser power. To obtain a first-order estimate of the relevant time scales, we employ a simplified yet effective approximation of the intrinsic thermal relaxation process.

For a circular drumhead, the theoretical thermal equilibrium time constant $\tau_{\mathrm{th}}$ is given by [15]:

$$\tau_{\mathrm{th}} = \frac{\rho_{\mathrm{gold}} C}{\kappa \left(2\mu_1 / a\right)^2}, \tag{S8}$$

where $\rho_{\mathrm{gold}}$, $C$, $\kappa$ denote the mass density, specific heat capacity, and thermal conductivity of the membrane material, respectively. $\mu_1$ = 2.4 is the first-order root of the Bessel function $J_0$ (considering that the fundamental mode exhibits the slowest decay rate and thus dominates the equilibration time). Using the material properties of ultrathin gold ($C$ = 129 J/(kg·K), $\kappa$ = 61.9 W/(m·K) [16]), taking $a$ = 5 μm as an example, the calculated characteristic time scale is ~ 44 ns.

Note that the thermal time constant is an intrinsic property of the structure, independent of the excitation mechanism. The nature of the thermal source (specifically, the laser spot size and flux distribution in our experiments) determines the initial temperature gradient and the final temperature maximum but takes no effect on the thermal decay rate. Although this analytical model assumes ideal conditions, it provides a reasonable order-of-magnitude estimate, and sets the lower limit. In the actual case, even if the actual relaxation were affected by other processes not included in the ideal case and became an order of magnitude slower, it would still remain in the nanosecond scale.

In addition to the above theoretical estimation, in order to gain additional understanding on the transient thermal behavior, we also conduct a finite element

modeling (FEM) simulation. The modeled geometry, illustrated in Figure S10, consists of a $SiO_2$ substrate, a suspended gold membrane, and the surrounding clamping substrate. We take the typical device dimensions used in our main text (5 μm diameter, 40 nm thickness) as an example.

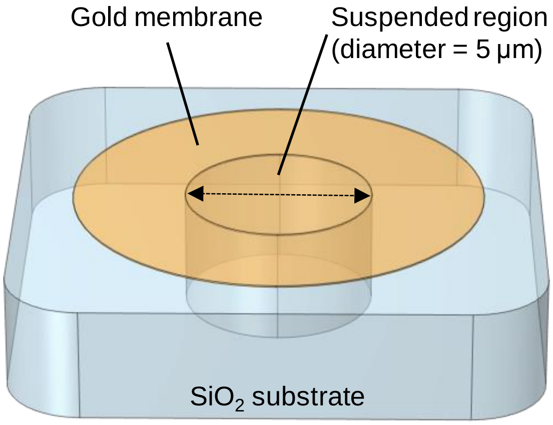


**Figure S10 Schematic of the geometry used in the FEM simulation.** Note that while the actual device sits on a nearly semi-infinite $SiO_2$ substrate, the substrate domain in the model is sufficiently large to act as an effective thermal bath.

The model incorporates the governing physical processes, including the laser source flux distribution (Gaussian-type), three-dimensional thermal conduction, and importantly, the interfacial thermal transport between the gold membrane and the $SiO_2$ substrate. The governing partial differential equation (PDE) is:

$$\begin{aligned} & r^2 = (x - x_0)^2 + (y - y_0)^2 \\ & \kappa\left(\frac{\partial^2 T}{\partial x^2} + \frac{\partial^2 T}{\partial y^2} + \frac{\partial^2 T}{\partial z^2}\right) + \frac{2P_0}{\pi t w^2}\exp\left(\frac{-2r^2}{w^2}\right) = 0, \quad (0 \le r \le r_{\mathrm{material}}) \\ & \boldsymbol{n}\left(\kappa\left(\frac{\partial^2 T}{\partial x^2} + \frac{\partial^2 T}{\partial y^2}\right)\right) - G_{\mathrm{B}}\Delta T(x, y) = 0, \quad (r_{\mathrm{suspended}} \le r \le r_{\mathrm{material}}) \end{aligned} \tag{S9}$$

where $r$ is the radial distance from any point ($x$, $y$) on the membrane to the laser spot center ($x_0$, $y_0$) (practically we try our best to locate the laser spot at the center of the membrane, hence $x_0 = y_0 = 0$ is used), $P_0$ is the laser power, $w = 0.7$ μm is the focused spot radius, and $t$ is the membrane thickness. $\Delta T(x, y)$ stands for the temperature difference in the supported aera, and $\boldsymbol{n}$ denotes the normal vector of the supported area facing $SiO_2$. In addition to the previously used parameter thermal conductivity $\kappa$,

we include the interfacial thermal conductance between gold and $SiO_2$ as $G_B$ = 61 MW/(m$^2$·K)[17].

We study the transient response over a 0–500 ns time window and plot the volume-averaged temperature as a function of time, with the results shown in Figure S11. A series of laser power level is considered (from 0.1 mW to 0.5 mW) in order to demonstrate that the equilibration speed is independent of the incident laser flux magnitude shed on the membrane.

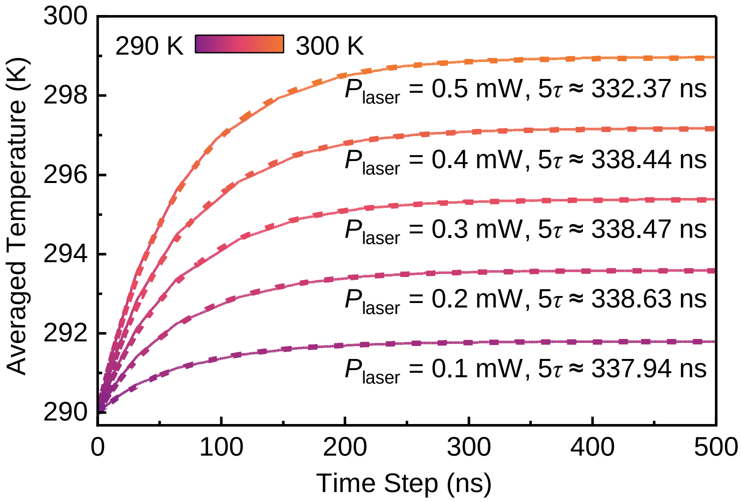


**Figure S11 Evolution of the volume-averaged temperature during the transient process.** Several different laser power levels are simulated. Solid lines are the FEM simulation results, dashed lines denote the corresponding fits to an exponential rise function. The thermal equilibrium time (to show a larger range of data we plot the temperature for a period of 5$\tau$, with time constant $\tau$ derived from the fit) is labeled below each curve.

Given that the temperature evolution follows a typical first-order step response, we fit the data by the following exponential rise function to extract the time constant:

$$T(t) = T_{\text{initial}} + \Delta T_{\text{eq}} \cdot \left(1 - e^{-t/\tau}\right) \qquad \text{(S10)}$$

where $t$ is the time variable, $T_{\text{initial}}$ is the ambient temperature (set to 290 K as per our lab environment), $\Delta T_{\text{eq}}$ is the total temperature rise at equilibrium, and $\tau$ denotes the time constant. We choose to plot a time period of 5$\tau$, which shows the process of establishing thermal equilibrium, corresponding to the temperature reaching within 1% ($e^{-5}$= 0.7%) of the final value. The fitting results for all the power levels (as labeled in

Fig. S11) yield a consistent equilibration time of ~340 ns.

Since the bolometric performance is intrinsically linked to the frequency shift and thus the device geometry (Section S4), it is essential to evaluate whether the thermal equilibration time depends on the membrane thickness. Qualitatively, while the membrane thickness directly influences the total heat capacity and the overall thermal conductance (and consequently the steady-state temperature rise), the thermal relaxation time is primarily governed by the material's lateral thermal conductivity and its lateral dimension. Given that the membrane thickness is orders of magnitude smaller than the lateral diameter, the vertical thermal gradient is negligible, and one can expect that the thickness should have a minimal impact on the characteristic time scale of thermal equilibration.

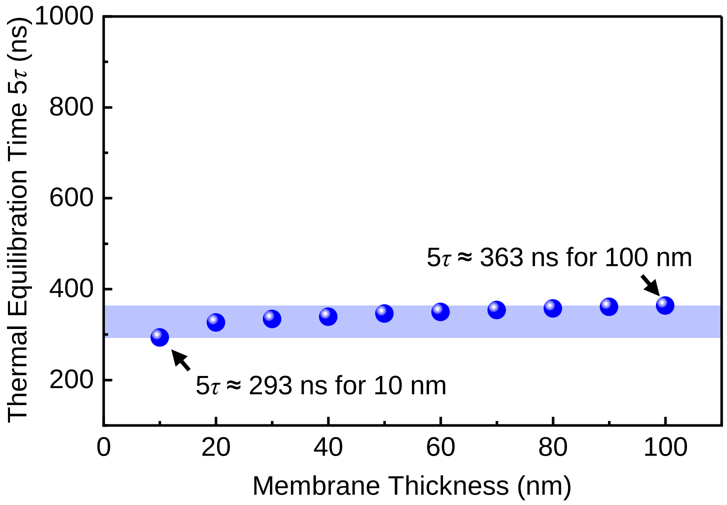


**Figure S12 Thermal equilibration time dependence on the membrane thickness.** The transient thermal responses for devices with thicknesses ranging from 10 – 100 nm are simulated and fitted to Equation S9. The derived equilibration time ($5\tau$) exhibits little variation across this typical thickness range.

To confirm this, we conduct additional FEM simulations to study the transient temperature evolution across a range of membrane thickness conditions. The resulting temperature profiles are fitted using Equation S10 to extract the corresponding time constants, as plotted in Figure S12. We consider a thickness range of 10–100 nm, representing typical dimensions for 2D NEMS resonators. The result reveals that the thermal equilibration time varies only slightly (within the 300–400 ns range) and

consistently remains on the order of hundreds of nanoseconds.

In all of our experiments, we modulate the laser power in a timescale of seconds (~3 s). Since this is orders of magnitude greater than the thermal equilibration time, one can safely assume that the device maintains a quasi-static thermal tension level throughout the measurement.

## S7. Theoretical Analysis of Device Elastic Behavior

The resonant frequency $f_{mn}$ of a fully-clamped circular drumhead resonator with tension can be expressed as [13]

$$f_{mn}=\left(\frac{k_2^{mn}a}{4\pi}\right)\sqrt{\frac{16D}{\sigma_{\text{gold}}a^4}\left[\left(\frac{k_2^{mn}a}{2}\right)^2+\frac{\gamma a^2}{4D}\right]}, \tag{S11}$$

where $\gamma$ (in N/m) is the surface tension of the membrane (because we use the minimal laser power, $\gamma_{\text{th}}$ can be negligible). The mode-dependent parameter ($k_2^{mn}a/2$) can be approximated by a double exponential function

$$\left(k_2^{mn}a/2\right)^2 \approx f(x)=\alpha+\left(\beta-\alpha\right)\exp\left\{-\eta\exp\left[\delta\ln\left(x\right)\right]\right\}, \tag{S12}$$

where $x = \gamma a^2/(4D)$, and $\alpha$, $\beta$, $\eta$ and $\delta$ are the mode-dependent values. For the fundamental frequency of the circular membrane resonator, $\alpha_0$ = 5.7832, $\beta_0$ = 10.215, $\eta_0$ = 1.1148 and $\delta_0$ = 0.4868. The maximum error in the modal approximation does not exceed 3.8%. As $x \to 0$, $f(x) \to \beta$ (ideal plate), and as $x \to \infty$, $f(x) \to \alpha$ (ideal membrane).

When $\gamma a^2/(4D) \to 0$, the surface tension becomes negligible, $(k_2^{mn}a/2)^2 \to \beta$. In this case, the resonator is mechanically in the "plate limit," and Equation S11 becomes:

$$f_{mn} = \frac{2\left(\frac{k_2^{mn}a}{2}\right)^2}{\pi a^2}\sqrt{\frac{D}{\sigma_{\text{gold}}}} \approx \frac{2\beta}{\pi}\sqrt{\frac{E_Y}{12\rho_{\text{gold}}\left(1-v^2\right)}}\frac{t}{a^2}. \tag{S13}$$

For the fundamental mode:

$$f_0 \approx \frac{2\beta_0}{\pi}\sqrt{\frac{E_Y}{12\rho_{\text{gold}}\left(1-v^2\right)}}\frac{t}{a^2}, \tag{S14}$$

from which we can observe that the frequency is proportional to $t/a^2$ when the device operates in the plate limit. Based on this observation, we fit the slope of the fundamental resonance of each device using Equation S14 to extract its Young's modulus $E_i$ (Figure 4(a) in the Main Text).

When $\gamma a^2/(4D) \rightarrow \infty$, the surface tension dominates, and the first term of Equation S11 can be neglected. $(k_2^{mn}a/2)^2 \rightarrow \alpha$. In this case, the resonator is mechanically in the "membrane limit," and Equation S11 becomes:

$$f_{mn} = \frac{\left(\frac{k_2^{mn}a}{2}\right)}{\pi a}\sqrt{\frac{\gamma}{\sigma_{\text{gold}}}} \approx \frac{\sqrt{\alpha}}{\pi a}\sqrt{\frac{\gamma}{\rho_{\text{gold}}t}}. \tag{S15}$$

For the fundamental mode:

$$f_0 \approx \frac{1}{\pi}\sqrt{\frac{\alpha_0\gamma}{\rho_{\text{gold}}}}\frac{1}{a\sqrt{t}}, \tag{S16}$$

from which we can observe that the frequency is proportional to $1/(at^{1/2})$ when the device operates in the membrane limit.

## S8. Extraction of Tension *γ* from Resonance Measurement

For a fully clamped circular drum, the fundamental-mode frequency described in Equation 2 of the main text consists of two components: the membrane term and the plate term

$$f_0 = \sqrt{f_{\mathrm{plate}}^2 + f_{\mathrm{membrane}}^2} \; . \tag{S17}$$

Using Equations S14 and S16, we calculate the frequencies corresponding to the plate part ($f_{\mathrm{plate}}$) and the membrane part ($f_{\mathrm{membrane}}$), while Equation 2 provides the total fundamental frequency ($f_0$). Figure S13(a) illustrates as device parameters vary, how the two terms contribute to $f_0$, and Figure S13(b) presents a two-dimensional plot showing the contribution of the plate term. The results reveal a clear trend: as the material thickness increases or the device diameter decreases, the dominant factor gradually shifts from the membrane term to the plate term.

To extract the surface tension $\gamma$, we select devices operating in the membrane and transition regimes and plot their data in Figure S13(c). The solid line represents the fitting result obtained from the frequency scaling law using Equation 2, with different values of tension $\gamma$. As shown in Figure S13(c), we can derive the initial tension of the devices from the measured frequency. The extracted tension $\gamma = 0.09 - 0.8$ N $m^{-1}$.

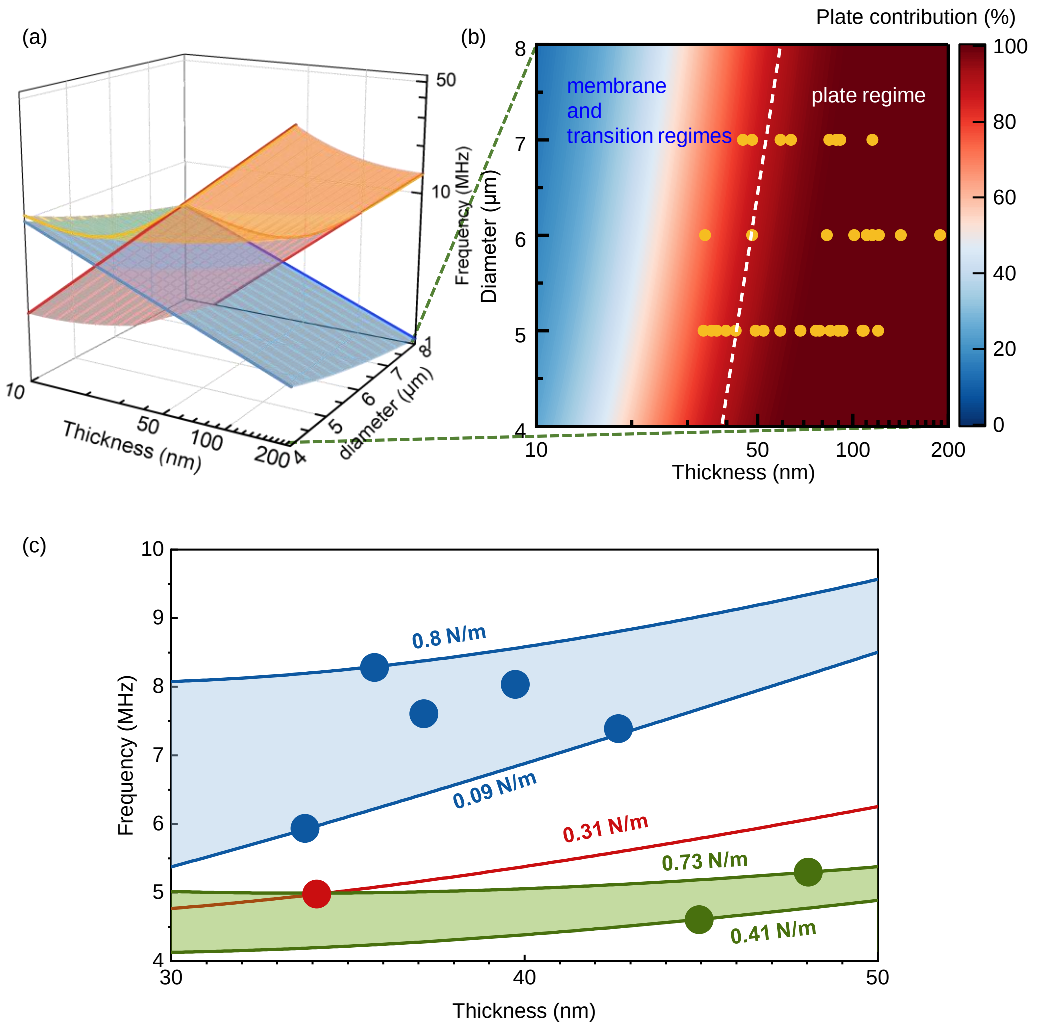


**Figure S13 Frequency scaling in gold circular drumhead resonators.** (a) Resonant frequency as a function of device thickness and diameter with $E_Y$ = 75.6 GPa and an in-plane tension of 0.5 N m$^{-1}$. The red and blue surfaces indicate the plate and membrane contributions, respectively. The curved yellow surface represents the device frequency, including contribution from both plate and membrane parts. (b) Contribution from the plate-term with varying thicknesses and diameters. White dashed line marks the boundaries of plate regime ($f_{plate}$ $\geq 0.9$ $f_0$), and yellow dots denote the measured devices. (c) Resonators operating in the membrane and transition regimes, showing the relationship between device thickness and fundamental-mode frequency. Solid lines represent the calculated frequencies with different tensions. Blue, red, and green curves/dots/areas correspond to devices with diameters $a$ = 5,6,7 μm, respectively.

## S9. Frequency Response of Different Devices

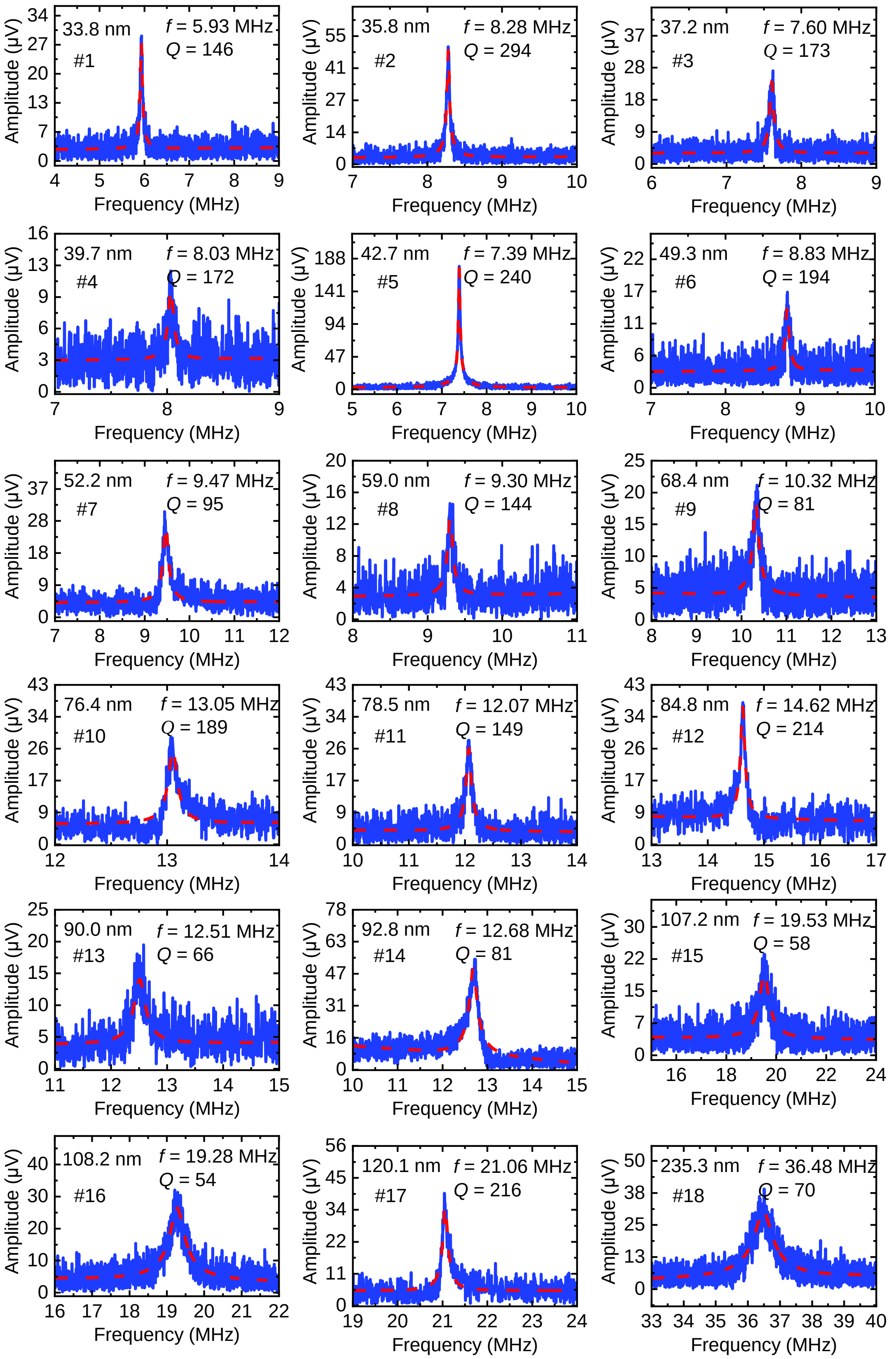


**Figure S14 Original data and fitting results for the resonance response of gold resonators with diameter of 5 μm.** Device resonance frequencies and quality factors are extracted from the fittings.

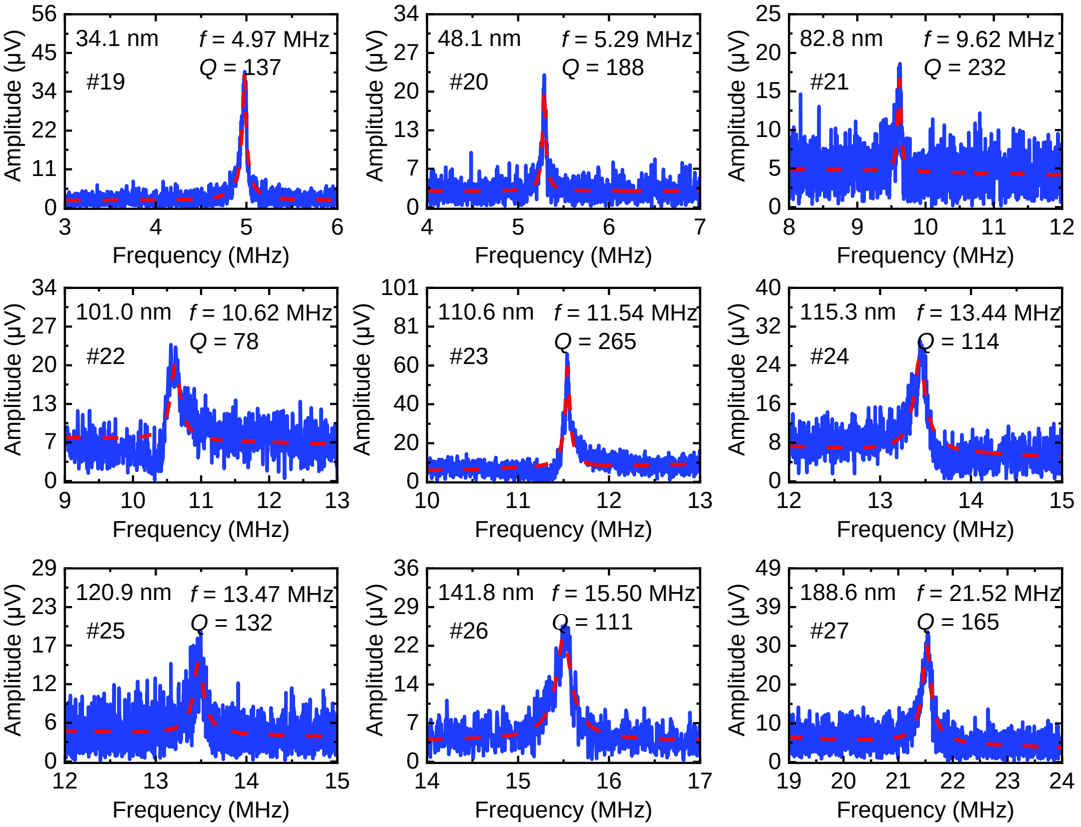


**Figure S15 Original data and fitting results for the resonance response of gold resonators with diameter of 6 μm.** Device resonance frequencies and quality factors are extracted from the fittings.

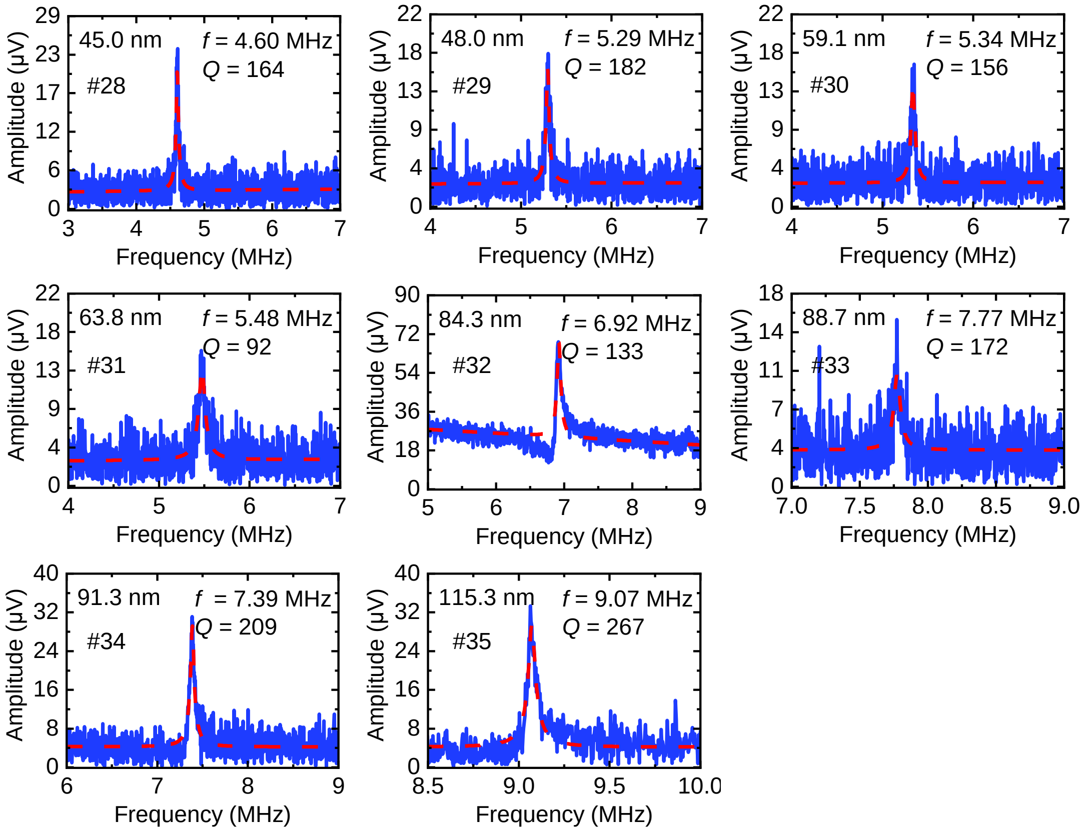


**Figure S16 Original data and fitting results for the resonance response of gold resonators with diameter of 7 μm.** Device resonance frequencies and quality factors are extracted from the fittings.

**Table S1 List of all measured gold resonators with device parameters.**

| Device # | Device Diameter (μm) | Thickness (nm) | Resonance Frequency $f_0$ (MHz) | Quality Factor $Q$ | Thickness /Diameter$^2$ $t/a^2$ ($m^{-1}$) | $f_0 \times Q$ (Hz) |
|---|---|---|---|---|---|---|
| 1 | 5 | 33.8 | 5.93 | 146 | 1352 | $8.66 \times 10^8$ |
| 2 | 5 | 35.8 | 8.28 | 294 | 1432 | $2.43 \times 10^9$ |
| 3 | 5 | 37.2 | 7.60 | 173 | 1488 | $1.31 \times 10^9$ |
| 4 | 5 | 39.7 | 8.03 | 172 | 1576 | $1.38 \times 10^9$ |
| 5 | 5 | 42.7 | 7.39 | 240 | 1708 | $1.77 \times 10^9$ |
| 6 | 5 | 49.3 | 8.83 | 194 | 1972 | $1.71 \times 10^9$ |
| 7 | 5 | 52.2 | 9.47 | 95 | 2088 | $9.00 \times 10^8$ |
| 8 | 5 | 59.0 | 9.30 | 144 | 2360 | $1.34 \times 10^9$ |
| 9 | 5 | 68.4 | 10.32 | 81 | 2736 | $8.36 \times 10^8$ |
| 10 | 5 | 76.4 | 13.05 | 189 | 3056 | $2.47 \times 10^9$ |
| 11 | 5 | 78.5 | 12.07 | 149 | 3140 | $1.80 \times 10^9$ |
| 12 | 5 | 84.8 | 14.62 | 214 | 3392 | $3.13 \times 10^9$ |
| 13 | 5 | 90.0 | 12.51 | 66 | 3600 | $8.26 \times 10^8$ |
| 14 | 5 | 92.8 | 12.68 | 81 | 3712 | $1.03 \times 10^9$ |
| 15 | 5 | 107.2 | 19.53 | 58 | 4288 | $1.13 \times 10^9$ |
| 16 | 5 | 108.2 | 19.28 | 54 | 4328 | $1.04 \times 10^9$ |
| 17 | 5 | 120.1 | 21.06 | 216 | 4804 | $4.55 \times 10^9$ |
| 18 | 5 | 235.3 | 36.48 | 70 | 9412 | $2.55 \times 10^9$ |

| Device # | Device Diameter (μm) | Thickness (nm) | Resonance Frequency $f_0$ (MHz) | Quality Factor $Q$ | Thickness /Diameter$^2$ $t/a^2$ (m$^{-1}$) | $f_0 \times Q$ (Hz) |
|---|---|---|---|---|---|---|
| 19 | 6 | 34.1 | 4.97 | 137 | 947 | $6.81 \times 10^8$ |
| 20 | 6 | 48.1 | 5.29 | 188 | 1336 | $9.95 \times 10^8$ |
| 21 | 6 | 82.8 | 9.62 | 232 | 2300 | $2.23 \times 10^9$ |
| 22 | 6 | 101.0 | 10.62 | 78 | 2806 | $8.28 \times 10^8$ |
| 23 | 6 | 110.6 | 11.54 | 265 | 3072 | $3.06 \times 10^9$ |
| 24 | 6 | 115.3 | 13.44 | 114 | 3203 | $1.53 \times 10^9$ |
| 25 | 6 | 120.9 | 13.47 | 132 | 3358 | $1.78 \times 10^9$ |
| 26 | 6 | 141.8 | 15.50 | 111 | 3939 | $1.72 \times 10^9$ |
| 27 | 6 | 188.6 | 21.52 | 165 | 5239 | $3.55 \times 10^9$ |
| 28 | 7 | 45.0 | 4.60 | 164 | 918 | $7.54 \times 10^8$ |
| 29 | 7 | 48.0 | 5.29 | 182 | 980 | $9.63 \times 10^8$ |
| 30 | 7 | 59.1 | 5.34 | 156 | 1206 | $8.33 \times 10^8$ |
| 31 | 7 | 63.8 | 5.48 | 92 | 1302 | $5.04 \times 10^8$ |
| 32 | 7 | 84.3 | 6.92 | 133 | 1720 | $9.20 \times 10^8$ |
| 33 | 7 | 88.7 | 7.77 | 172 | 1810 | $1.34 \times 10^9$ |
| 34 | 7 | 91.3 | 7.39 | 209 | 1863 | $1.54 \times 10^9$ |
| 35 | 7 | 115.3 | 9.07 | 267 | 2353 | $2.42 \times 10^9$ |

**Table S2 Comparison of responsivity and linearity for previously reported MEMS/NEMS bolometers and the present device.**

| Ref. | Year | Materials | Scale | Laser (nm) | \|Responsivity\| (Hz/μW) | \|Normalized Responsivity\| (ppm/mW) |
|---|---|---|---|---|---|---|
| 18 | 2022 | $Si_3N_4$ | 300 nm thick; 200 × 200 μm plate; 560 × 20 μm tethers | 532 | 0.6 | |
| 19 | 2023 | Polyimide/ carbon | 3 × 3 mm | 808 | | 2.2 |
| 20 | 2009 | Quartz | Diameter: 1 mm; Thickness: ~18 μm | 2000-20000 | 14.3 | |
| 21 | 2014 | GaN-on-SOI | Length: 30 μm; Width: 5 μm; Thickness: 1.425 μm | Near-IR | 1.7 | 0.0168 |
| 22 | 2022 | MXene/ $LiNbO_3$ | 2 mm × 1.3 mm × 35 μm | 830 | 15.28 | |
| 23 | 2011 | Quartz | Thickness: 100 μm Diameter:200 μm | Near-IR | 11.4 | |
| 24 | 2023 | MXene/ $LiNbO_3$ | 8400 μm × 1000 μm | 830 | 10.42 | |
| 25 | 2025 | Phononic Crystal /Lithium Niobate | 384 μm × 78 μm | 6300 | 136 | |
| 26 | 2020 | Black Phosphorus | Thickness: 30 nm; Diameter: 4.6 μm | 785 | 310 | |
| **This work** | **2025** | **Au** | **Thickness: 39.7 nm Diameter: 5 μm** | **633** | **114** | |

## References

[1] Zhu, J. *et al.* Achieving 1.2 fm/Hz $^{1/2}$ Displacement Sensitivity with Laser Interferometry in Two-Dimensional Nanomechanical Resonators: Pathways towards Quantum-Noise-Limited Measurement at Room Temperature. *Chinese Phys. Lett.* **40**, 038102 (2023).

[2] Blake, P. *et al.* Making graphene visible. *Appl. Phys. Lett.* **91**, 063124 (2007).

[3] Wang, Z. & Feng, P. X.-L. Interferometric Motion Detection in Atomic Layer 2D Nanostructures: Visualizing Signal Transduction Efficiency and Optimization Pathways. *Sci Rep* **6**, 28923 (2016).

[4] Yakubovsky, D. I., Arsenin, A. V., Stebunov, Y. V., Fedyanin, D. Yu. & Volkov, V. S. Optical constants and structural properties of thin gold films. *Opt. Express* **25**, 25574 (2017).

[5] Malitson, I. H. Interspecimen Comparison of the Refractive Index of Fused Silica*,†. *J. Opt. Soc. Am.* **55**, 1205 (1965).

[6] Handbook of Optical Constants of Solids.

[7] Cleland, A. N. *Foundations of Nanomechanics: From Solid-State Theory to Device Applications*. (Springer, Berlin Heidelberg, 2003). doi:10.1007/978-3-662-05287-7.

[8] Lee, J., Wang, Z., He, K., Shan, J. & Feng, P. X.-L. High Frequency $MoS_2$ Nanomechanical Resonators. *ACS Nano* **7**, 6086–6091 (2013).

[9] Wang, Z. & Feng, P. X.-L. Design of black phosphorus 2D nanomechanical resonators by exploiting the intrinsic mechanical anisotropy. *2D Mater.* **2**, 021001 (2015).

[10] Zhu, J. *et al.* Frequency Scaling, Elastic Transition, and Broad-Range Frequency Tuning in $WSe_2$ Nanomechanical Resonators. *Nano Lett.* **22**, 5107–5113 (2022).

[11] Wang, L. *et al.* Investigating thermal properties of 2D non-layered material using a NEMS-based 2-DOF approach towards ultrahigh-performance bolometer. *National Science Review* **11**, nwae248 (2024).

[12] Tanimoto, H., Sakai, S. & Mizubayashi, H. Mechanical property of high density nanocrystalline gold prepared by gas deposition method. *Nanostructured Materials* **12**, 751–756 (1999).

[13] Suzuki, H., Yamaguchi, N. & Izumi, H. Theoretical and experimental studies on the resonance frequencies of a stretched circular plate: Application to Japanese drum diaphragms. *Acoust. Sci. & Tech.* **30**, 348–354 (2009).

[14] Oliva, A. I., Comparán-Rodriguez, G. G., Sosa, V. & Oliva-Avilés, A. I. Poisson’s ratio determination of Au nanofilms by piezoresistive measurements. *J Mater Sci* **58**, 8563–8571 (2023).

[15] Reichenbach, R. B. *et al.* Resistively actuated micromechanical dome resonators. in (eds

Janson, S. W. & Henning, A. K.) 51 (San Jose, CA, 2004). doi:10.1117/12.524175.

[16] Lin, H., Xu, S., Li, C., Dong, H. & Wang, X. Thermal and electrical conduction in 6.4 nm thin gold films. *Nanoscale* **5**, 4652 (2013).

[17] Hatam-Lee, S. M., Jabbari, F. & Rajabpour, A. Interfacial thermal conductance between gold and $SiO_2$ : A molecular dynamics study. *Nanoscale and Microscale Thermophysical Engineering* **26**, 40–51 (2022).

[18] Vicarelli, L., Tredicucci, A. & Pitanti, A. Micromechanical Bolometers for Subterahertz Detection at Room Temperature. *ACS Photonics* **9**, 360–367 (2022).

[19] Yao, J., Yao, Y. & Liu, T. “Fire-beetles” inspired all-solid infrared sensor with bolometric and photomechanic dual sensing modes. *Mater. Des.* **230**, 111968 (2023).

[20] Kao, P. & Tadigadapa, S. Micromachined quartz resonator based infrared detector array. *Sensors Actuat. A-Physical* **149**, 189–192 (2009).

[21] Gokhale, V. J. & Rais-Zadeh, M. Uncooled Infrared Detectors Using Gallium Nitride on Silicon Micromechanical Resonators. *Journal of Microelectromechanical Systems* **23**, 803–810 (2014).

[22] Feng, L. *et al.* Study of MXene Ti3C2Tx quantum dot thin film on quartz bulk acoustic wave uncooled infrared sensors. *Opt Express* **30**, 34129–34139 (2022).

[23] Pisani, M. B., Ren, K., Kao, P. & Tadigadapa, S. Application of Micromachined Y -Cut-Quartz Bulk Acoustic Wave Resonator for Infrared Sensing. *Journal of Microelectromechanical Systems* **20**, 288–296 (2011).

[24] Feng, L. *et al.* High-sensitivity non-cooled near-infrared detector based on lithium niobate surface acoustic wave resonators combined with MXene $Ti_3C_2Tx$ quantum dot thin films. *Opt. Express, OE* **31**, 25829–25839 (2023).

[25] Xi, Z. *et al.* Room-Temperature Mid-Infrared Detection Using Metasurface-Absorber-Integrated Phononic Crystal Oscillator. *Laser & Photonics Reviews* **19**, e00498 (2025).

[26] Islam, A., Lee, J. & Feng, P. X.-L. Black Phosphorus NEMS Resonant Infrared (IR) Detector. in *2020 IEEE 33rd International Conference on Micro Electro Mechanical Systems (MEMS)* 826–829 (2020). doi:10.1109/MEMS46641.2020.9056366.
